# Multidrug Resistance in Cancer: Understanding Molecular Mechanisms, Immunoprevention and Therapeutic Approaches

**DOI:** 10.3389/fonc.2022.891652

**Published:** 2022-06-23

**Authors:** Talha Bin Emran, Asif Shahriar, Aar Rafi Mahmud, Tanjilur Rahman, Mehedy Hasan Abir, Mohd. Faijanur - Rob Siddiquee, Hossain Ahmed, Nova Rahman, Firzan Nainu, Elly Wahyudin, Saikat Mitra, Kuldeep Dhama, Mahmoud M. Habiballah, Shafiul Haque, Ariful Islam, Mohammad Mahmudul Hassan

**Affiliations:** ^1^ Department of Pharmacy, BGC Trust University Bangladesh, Chittagong, Bangladesh; ^2^ Department of Pharmacy, Faculty of Allied Health Sciences, Daffodil International University, Dhaka, Bangladesh; ^3^ Department of Immunology and Microbiology, School of Medicine, University of Texas Rio Grande Valley, McAllen, TX, United States; ^4^ Department of Biochemistry and Molecular Biology, Mawlana Bhashani Science and Technology University, Tangail, Bangladesh; ^5^ Department of Biochemistry and Molecular Biology, Faculty of Biological Sciences, University of Chittagong, Chittagong, Bangladesh; ^6^ Faculty of Food Science and Technology, Chattogram Veterinary and Animal Sciences University, Chattogram, Bangladesh; ^7^ Department of Biochemistry and Molecular Biology, University of Dhaka, Dhaka, Bangladesh; ^8^ Department of Biotechnology and Genetic Engineering, University of Development Alternative, Dhaka, Bangladesh; ^9^ Department of Biochemistry and Molecular Biology, Jahangirnagar University, Dhaka, Bangladesh; ^10^ Department of Pharmacy, Faculty of Pharmacy, Hasanuddin University, Makassar, Indonesia; ^11^ Department of Pharmacy, Faculty of Pharmacy, University of Dhaka, Dhaka, Bangladesh; ^12^ Division of Pathology, ICAR-Indian Veterinary Research Institute, Bareilly, India; ^13^ Medical Laboratory Technology Department, Jazan University, Jazan, Saudi Arabia; ^14^ SMIRES for Consultation in Specialized Medical Laboratories, Jazan University, Jazan, Saudi Arabia; ^15^ Research and Scientific Studies Unit, College of Nursing and Allied Health Sciences, Jazan University, Jazan, Saudi Arabia; ^16^ Bursa Uludağ University Faculty of Medicine, Bursa, Turkey; ^17^ EcoHealth Alliance, New York, NY, United States; ^18^ Queensland Alliance for One Health Sciences, School of Veterinary Science, The University of Queensland, Gatton, QLD, Australia; ^19^ Department of Physiology, Biochemistry and Pharmacology, Faculty of Veterinary Medicine, Chattogram Veterinary and Animal Sciences University, Chattogram, Bangladesh

**Keywords:** multidrug resistance, cancer, immuno-prevention, microRNA, intracellular and extracellular ATP

## Abstract

Cancer is one of the leading causes of death worldwide. Several treatments are available for cancer treatment, but many treatment methods are ineffective against multidrug-resistant cancer. Multidrug resistance (MDR) represents a major obstacle to effective therapeutic interventions against cancer. This review describes the known MDR mechanisms in cancer cells and discusses ongoing laboratory approaches and novel therapeutic strategies that aim to inhibit, circumvent, or reverse MDR development in various cancer types. In this review, we discuss both intrinsic and acquired drug resistance, in addition to highlighting hypoxia- and autophagy-mediated drug resistance mechanisms. Several factors, including individual genetic differences, such as mutations, altered epigenetics, enhanced drug efflux, cell death inhibition, and various other molecular and cellular mechanisms, are responsible for the development of resistance against anticancer agents. Drug resistance can also depend on cellular autophagic and hypoxic status. The expression of drug-resistant genes and the regulatory mechanisms that determine drug resistance are also discussed. Methods to circumvent MDR, including immunoprevention, the use of microparticles and nanomedicine might result in better strategies for fighting cancer.

## 1 Introduction

Cancer is an emerging and rarely curable disease, and nearly two million new cases of cancer were diagnosed in 2020, increasing the overall burden on society (1). The identification of both affordable and efficient cancer treatments remains an important goal for both researchers and clinicians. Currently, chemotherapy is viewed as one of the most promising cancer treatments modalities for reducing the cancer burden. However, chemotherapy fails in nearly 90% of cases because tumor cells develop resistance against the anticancer agent, resulting in increased cancer invasion the progression to metastases, which increases the difficulty of treating cancer effectively (2). Depending on the cancer type, some preventive measures and treatments are not readily available in developing countries, and even developed countries experience difficulties with cancer treatments due to the increasing development of resistance against chemotherapeutic agents and targeted therapies. Several factors, including genetics, micro RNAs (miRNAs), and long noncoding RNAs (lncRNAs), contribute to the development of multidrug resistance (MDR) in cancer cells (3–6). MDR genes play significant roles in the development of drug resistance. Studies have identified four genes in the MDR family, including two genes (*MDR1* and *MDR2*) expressed in humans. *MDR1* (also known as *ABCB1* or ATP Binding Cassette Subfamily B Member 1) encodes P-glycoprotein (P-gp), a Ca^2+^-dependent efflux pump that has been associated with the development of resistance against anthracyclines, vinca alkaloids, actinomycin D, and paclitaxel resistance (7–9). Using *MDR* cDNAs, a gene transfer experiment examined the effects of enhanced P-gp expression under the control of various eukaryotic promoters, which introduced MDR in cultured cells previously sensitive to chemotherapeutic agents (10, 11).

Understanding the molecular mechanisms that result in the development of drug resistance is an increasingly important issue, which has been approached through the comprehensive genomics analysis of MDR cancer cells, including the epigenetics associated with drug resistance and the identification of MDR genes. Certain conditions, such as hypoxia and autophagy, in cancer cells are also known to contribute to drug resistance and reduced drug efficacy (12–15). According to a World Health Organization (WHO) report from 2019, cancer is currently the second-leading cause of death worldwide. Globally, an estimated 9600 thousand deaths are attributed to cancer worldwide, representing 1 in every 6 deaths (16). Many cancer treatment mechanisms have been developed, and drug-sensitive cancer cells can be killed using conventional chemotherapeutic anticancer agents, which typically act by causing DNA damage using highly toxic and non-specific mechanisms (17, 18). However, to overcome drug resistance in cancer cells, the identification of drugs that can be delivered to specific molecular targets is necessary to improve the specificity and precision of the treatment. Several ongoing studies are exploring potentially effective anticancer drugs (19–21). Although many anticancer drugs exhibit remarkable efficacy during primary treatment, drug resistance often develops in many cancer patients as treatment progresses (19, 21). Studies have found that 30%–55% of patients with non–small cell lung cancer (NSCLC) experience relapse, followed by death (22). Another study reported that one year after surgery, and associated chemotherapy, 50%–70% of ovarian adenocarcinomas recur (22, 23). Approximately 20% of pediatric acute lymphoblastic leukemia cases recur (24).

Immunoprevention is another outstanding potential approach for cancer treatment, including MDR cancer (25), based on the activation of the patients’ immune systems. Preventive vaccines are the most successful approaches for cancer prevention, but other agents have been explored, including immunomodulators and antibodies. Immunoprevention aims to prevent cancer development, and studies are ongoing to determine the potential for applying the underlying mechanism of Immunoprevention to cancer types that are not associated with infectious agents (26). Studies exploring the limitations of Immunoprevention strategies for cancer treatment have revealed that MDR represents a common limitation across all cancer treatment modalities. Understanding the mechanisms that underlie the development of MDR in cancer may identify potential strategies for overcoming this limitation, improving the efficacy of cancer treatments. Some alternative approaches are also being explored, such as blocking the activity of cancer-derived microparticles (MPs), the use of nanoparticles for the targeted delivery of anticancer drugs, the development of nanomedicines, and the use of clustered regularly interspaced short palindromic repeats (CRISPR)/CRISPR-associated (Cas)9 technology to overcome the development of MDR (27–29). This review highlights several mechanisms that lead to MDR development, including the role of epigenetics, in addition to MDR regulators and mutational effects. This review also provides an overview of current approaches and advancements in the fight against MDR, including the identification of MDR biomarkers, Immunoprevention and its limitations, alternative therapeutic approaches, and treatment-related risk factors for the development of drug resistance in cancer. This review will provide future researchers with a comprehensive update on the current state of research regarding MDR in cancer.

## 2 Multidrug Resistance in Cancer

**Figure 1:**
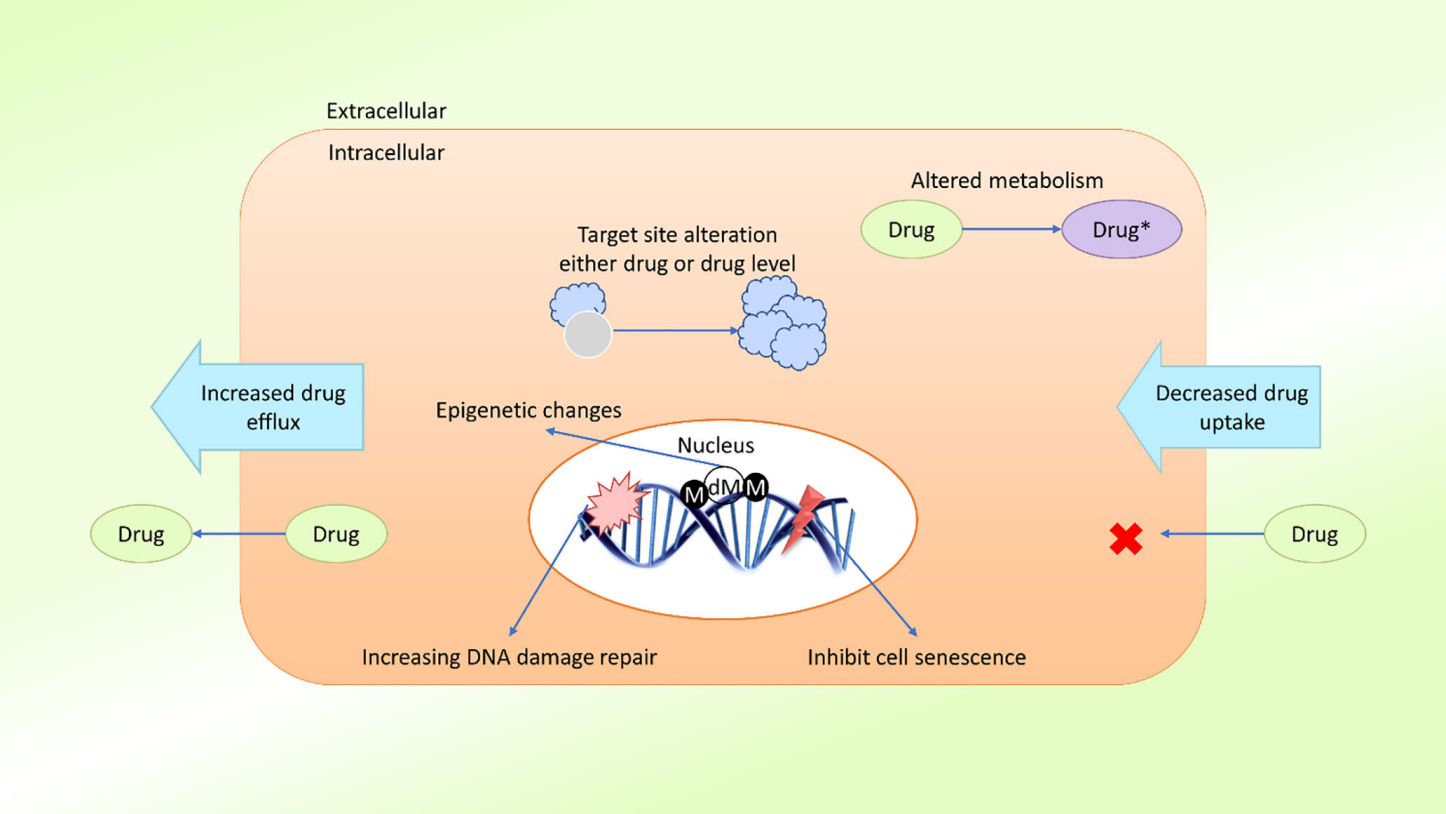
Schematic presentation of possible drug resistance mechanisms in cancer. Cancer cells develop resistance to anticancer agents (drugs) through various mechanisms, such as diminished drug uptake, enhanced drug efflux, improved DNA damage repair, resistance to cellular senescence (apoptosis suppression), alteration of drug metabolism, alteration of the drug target, epigenetic changes, and target gene amplification. These mechanisms act either individually or in combination, leading to the development of single or multidrug resistance in cancer cells (M, methylation; dM, demethylation).

In the field of cancer treatment, MDR is defined as the ability of cancer cells to survive treatment with a variety of anticancer drugs (30), similar to the concept commonly applied to antibiotic treatment. Cancer patients can be treated with two types of treatment: local and systemic. Radiation and surgery are considered local treatments, whereas chemotherapy, hormone therapy, and targeted therapy are considered systemic treatments (31). Systemic treatments are especially effective against metastatic or late phase cancers. Growing evidence suggests that MDR is mediated by the increased efflux of chemotherapeutic drugs, which reduces the drug absorption by cancer cells (32). The mechanism of MDR may also be mediated by the release of drugs outside of the cells. MDR may develop due to oncogene mutations, changes in the tumor microenvironment (TME), tumor heterogeneity, target site mutations, or epigenetic changes (33, 34) (**Figure 1**).

### 2.1 Characterization of Resistance in Cancer

**Figure 2:**
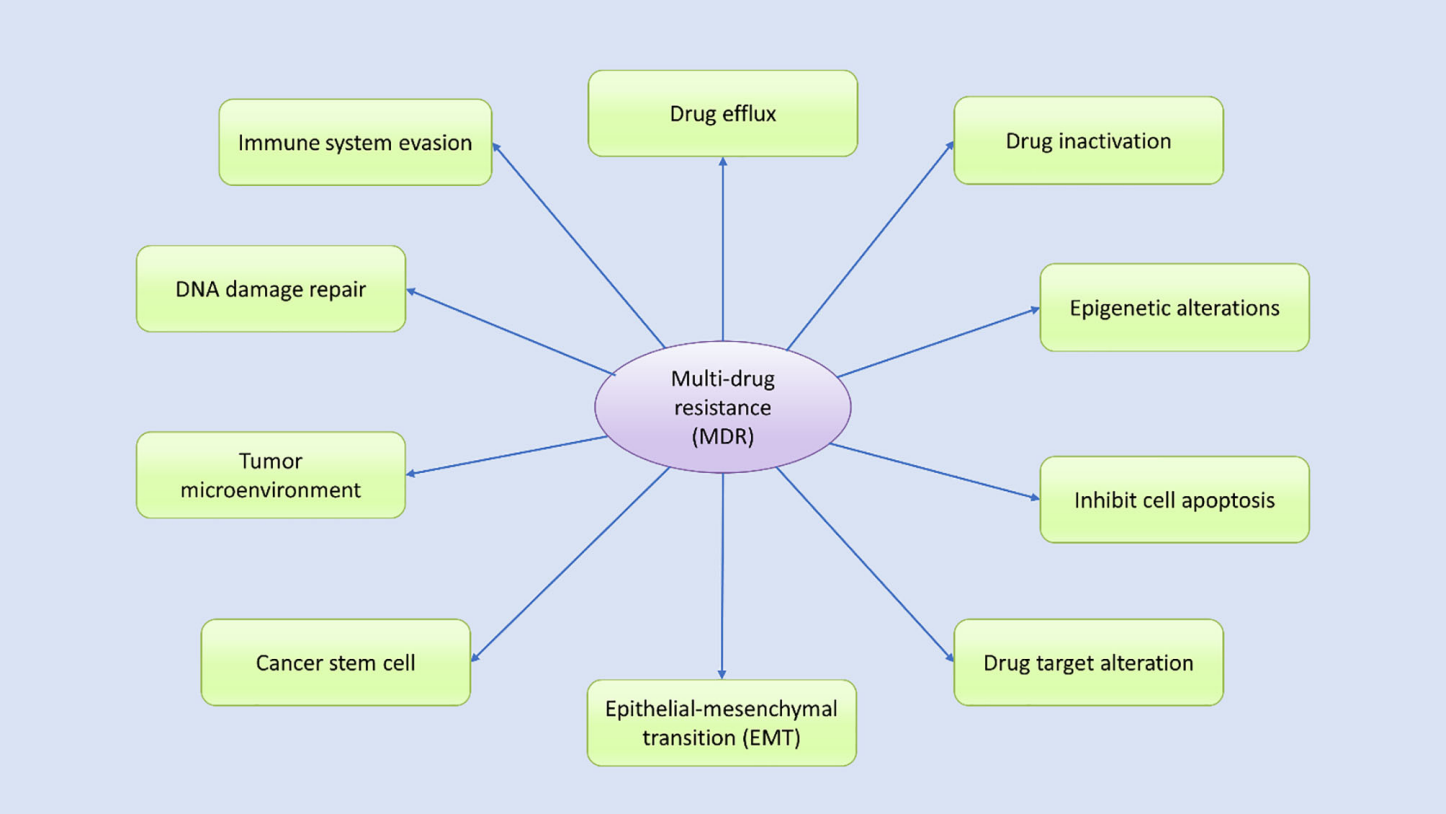
Various potential mechanisms contribute to multidrug resistance. Many internal and external factors have been associated with the development of multidrug resistance in human cancer cells through either direct or indirect effects. Drug efflux, changes in cellular drug levels, drug inactivation, altered epigenetic states, epithelial–mesenchymal transition (EMT), the tumor microenvironment, DNA damage repair, cancer stem cell propagation, and immune system evasion are well-studied mechanisms thought to contribute to MDR through various signal transduction pathways, either independently or in combination.

Drug resistance in cancer is an intimate occurrence that results when cancer becomes tolerant of pharmaceutical treatment (18). An extensive range of factors contributes to the development of resistance against anticancer drugs, including genetic mutations, altered epigenetics, enhanced drug efflux, and various changes in other molecular and cellular mechanisms, including the activation of specific signaling pathways (18, 35–37). Growing evidence suggests that to effectively treat cancer patients, the mechanisms underlying the development of drug resistance in patients must be analyzed (30, 38). During the progression of cancer treatment, the risk of MDR increases over time (30). Cancer cells evolve daily to manage insults and survive, which can make cancer treatment challenging (18, 22, 30, 32). Understanding the biochemical and genetic aspects that contribute to MDR in cancer may improve drug design, leading to the development of novel treatment options for cancer patients (39). Multiple potential mechanisms have been identified by various studies as contributing to MDR, which can be categorized according to their features (**Figure 2**). Drug resistance can occur due to the activation of both intrinsic (pre-existing) or acquired (induced by drugs) mechanisms, and both types of factors play significant roles in the development of drug resistance.

#### 2.1.1 Intrinsic and Acquired Drug Resistance

Intrinsic resistance refers to pre-existing resistance mechanisms present in a patient prior to drug administration, resulting in reduced treatment potency. Studies suggest that intrinsic resistance can be caused by (a) inherited genetic alterations that result in most of the tumor cells having reduced responses to chemotherapy and target drugs; (b) unresponsive subpopulations, such as cancer stem cells, which determine tumor diversity and can reduce the efficacy anticancer agents; or (c) anticancer drug removal through the activation of intrinsic pathways (40). Cancer cell proliferation and programmed cell death-related genetic alterations may also contribute to intrinsic drug resistance in cancer cells (40). Intrinsic resistance decreases the initial efficacy of drug treatment, independent of any prior exposure to the therapeutic agent (41). Inherent genetic mutations, such as those found in triple-negative breast cancer cells; tumor heterogeneity; and pre-existing subpopulations, cells in which intrinsic pathway activation promotes proliferation and the presence of cancer stem cells, serve as a defense barrier against the toxicity of anticancer drugs, contributing to intrinsic drug resistance (41, 42). For example, intrinsic cisplatin resistance was identified in gastric cancer patients with human epidermal growth factor receptor 2 (HER2) overexpression (43). HER2 overexpression upregulates the Snail transcription factor, triggering morphologic changes analogous to the epithelial–mesenchymal transition (EMT), resulting in cancer cell resistance against cisplatin therapy (43, 44). In addition, HER2/Snail double-positive patients have an even lower cisplatin response rate than single-positive patients (41). A team of researchers showed that Slug and Snail mediated EMT and promoted self-renewal and resistance to p53-induced programmed cell death (44).

Genotypic alterations comprise mutations, chromosomal rearrangements, gene amplifications, transposable elements, gene deletions, gene translocations, and miRNA modifications, and genomic instability in cancer can lead to intercellular genetic heterogeneity (18). In addition, epigenetic factors involving mRNA, transcriptomic, and proteomic heterogeneity can also be affected by genotypic alterations (42). Genetic differences can also be reflected by differences in the cell cycle, non-specific dissimilarities among cells, or ordered arrangements of cells under cancer stem cell theory (36, 45, 46). Eventually, these changes manifest as tumor heterogeneity, which is considered to represent the combination of intrinsic factors. Extrinsic factors can also affect the response to treatment, including pH, hypoxia, and paracrine signaling interactions between stromal and other tumor cells (47, 48). Acquired resistance refers to the reduction in anticancer agent potency following repeated drug administrations. Acquired resistance can be induced by (a) the activation of a second proto-oncogene, which serves as a newly occurring driver gene; (b) the modification of drug targets to reduce recognition; and (c) changes in the TME (41). Novel genetic mutations can induce resistance and regeneration in previously consolidated tumors. A genomic study showed a discrepancy in eight AML patients, revealing that novel genetic mutations are responsible for tumor resistance and regeneration (49). New mutations or altered expression patterns were associated with the development of acquired resistance against targeted therapy. Imatinib, a tyrosine kinase inhibitor (TKI) that targets BCR-ABL, is typically used to treat chronic myeloid leukemia, and 20%–30% of patients develop resistance after treatment (50). The secondary T315I point mutation that develops in BCR-ABL is believed to be an underlying mechanism of acquired resistance (50, 51). Chemotherapeutic drugs are cytotoxic to cancerous cells, causing DNA damage that likely increases the rate of novel mutations (41). The TME has also been associated with acquired chemoresistance. Cancer cells release exosomes carrying miRNAs that are used to communicate with tumor-associated macrophages and other cancer cells, creating a link between the TME and cancer cells (52).

#### 2.1.2 ATP and ATP-Mediated Drug Resistance

Two types of ATP exist in the body, intracellular and extracellular, both of which play crucial roles in cancer cell survival, growth, and resistance (53). ATP acts as a biological currency and plays a significant and necessary role in the survival and development of both cancer cells and normal cells in the body (54). One study reported that cancer cells have higher intracellular ATP levels are than normal cells due to a phenomenon known as the Warburg effect, in which cancer cells display enhanced glucose uptake and aerobic glycolysis, resulting in increased ATP production (55, 56). Moreover, cancer cells with acquired resistance are present with even higher intracellular ATP levels than their parental cell lines (31, 57). Thus, ATP and ATP-mediated transporters and signaling pathways are thought to play influential roles in the development of drug resistance.

##### 2.1.2.1 Intracellular ATP Promotes Drug Resistance

Intracellular ATP levels vary between cancer cells and normal cells due to the Warburg effect (56, 58). In colon cancer cell lines, the ATP levels in chemo-resistant cell lines were two-fold higher than those in their drug-sensitive parental cell lines (59). The ATP-binding cassette (ABC) transporter families are well-known ATP-dependent transporters that move nutrients and soluble compounds throughout the cell. Based on the pattern of substrate translocation, ABC transporters are classified into importers or exporters (60). ABC exporters and importers have been shown to have the same transport process, due to their structural likenesses (61). The core of all ABC transporters (both ABC importers and exporters) consists of the following components: two nucleotide binding domains (NBDs), and two transmembrane domains (TMDs) or membrane spanning domains (MSDs). Additionally, there are a number of extracellular soluble substrate binding domains (SBDs) in some ABC importers, which are not required by the ABC exporters (62). MSDs are usually responsible for substrate identification and translocation, while NBDs are responsible for ATP binding and hydrolysis (61). Nonetheless, the SBDs of ABC importers on the extracellular portion helps in capturing and delivering the transported substrate to the MSDs (61). A recent study identified three members in the ABC transporter family: P-gp, multidrug resistance protein 1 (MRP1)/ATP Binding Cassette Subfamily C Member 1 (*ABCC1*), and breast cancer resistance protein (BCRP)/ABC subfamily G member 2 (*ABCG2*) (63). P-gp is a well-known multidrug membrane transporter that transports chloride outside of the cell, where it binds with a wide range of chemotherapy agents (e.g., doxorubicin [DOX], vinblastine, and taxol). After binding with chemotherapeutic drugs, ATP becomes hydrolyzed, resulting in an alteration in the P-gp structure, releasing the drug into the extracellular space. The transporter returns to its initial structural conformation through a second ATP hydrolysis step, causing drug efflux (64, 65). The application of a glycolysis inhibitor to diminish intracellular ATP levels can alert resistant cancer cells (57). Research has reported that increased energy storage is necessary for treatment-resistant cell lines, protecting them from environmental stress and xenobiotics. In addition, intracellular ATP plays a metabolic role in the acquired resistance against chemotherapy drugs. Therefore, a necessary condition for resistant cancer cells is elevated levels of intracellular ATP (41, 56). Another study reported that in ovarian adenocarcinoma cells, cisplatin resistance was associated with increased intracellular ATP levels (57). These studies suggest that the metabolic contributions of intracellular ATP have significant impacts on acquired drug resistance against chemotherapeutic drugs.

##### 2.1.2.2 Extracellular ATP Promotes Drug Resistance

**Figure 3:**
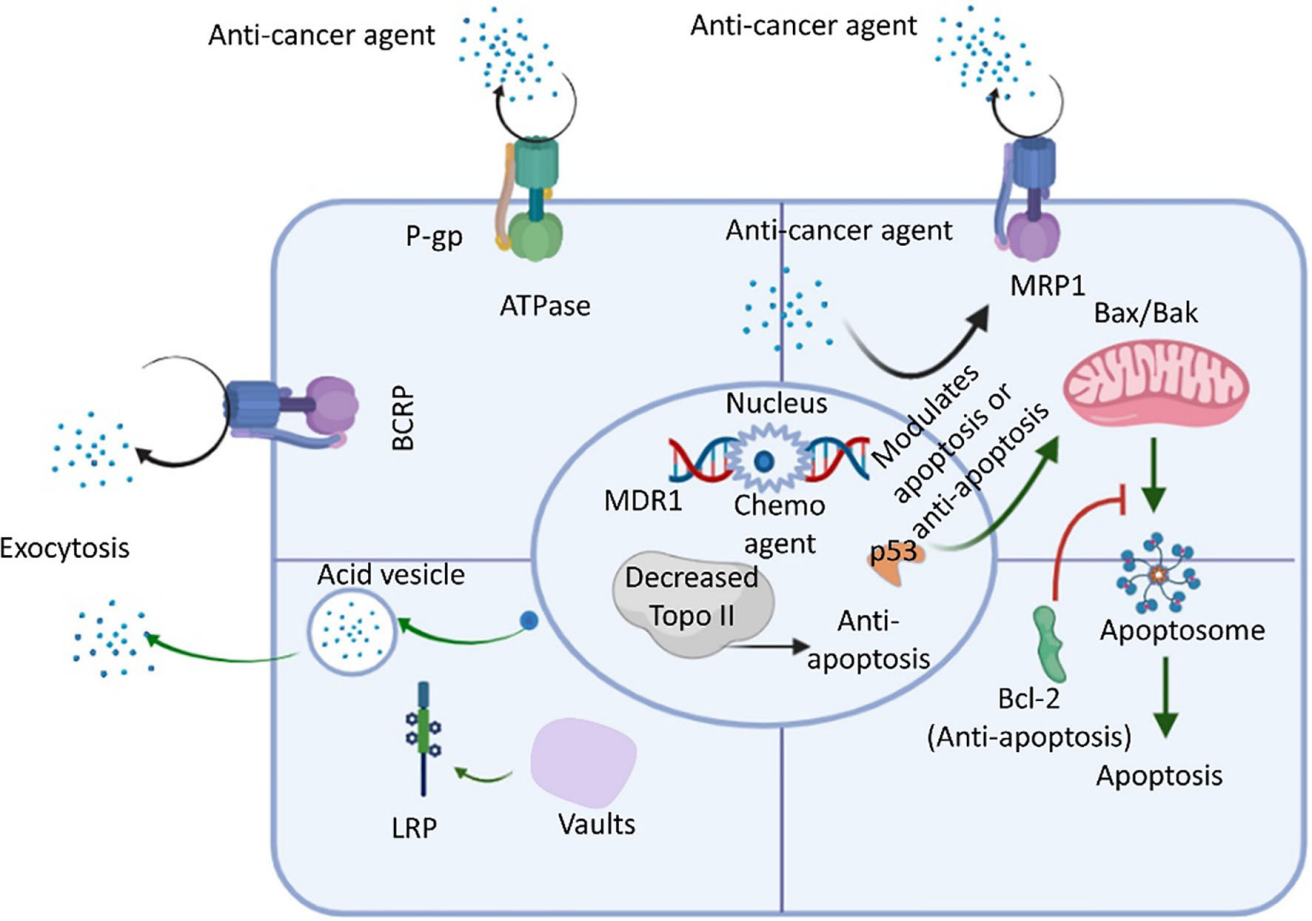
An overview of drug resistance mechanisms in cancer cells using ABC transporter, LRP, *Bcl-2*, and *Topo ll*. The ATP-binding cassette (ABC) transporter is an ATP-activated transporter. In general chemotherapy, cells express ABC transporters to remove foreign molecules (e.g., xenobiotics, anticancer agents, etc.) from the intracellular environment. P-glycoprotein (P-gp), multidrug-resistant protein 1 (MRP-1), and breast cancer resistance protein (BCRP) are the predominant members of the ABC transporter family. Lung resistance protein (LRP) resides in vaults (cytoplasmic) and contributes to the exocytosis of foreign molecules, including anticancer drugs. Research also revealed that the upregulation of *bcl-2* (an anti-apoptotic factor acted upon by anticancer agents that activate the normal apoptosis process), p53 loss-of-function of p53, and the downregulation of *topoisomerase II* (*Topo-II*) also decrease cell apoptosis to increase the resistance of cancer cells to anticancer drugs (74).

Growing evidence suggests that extracellular ATP levels could vary by 10^3^–10^4^ times in various cancer cells compared with normal tissues (54, 56, 66). One study examined the effects of eight cancer agents, including drugs used for targeted therapy and available chemotherapeutic drugs, in five cancer cell lines originating from different organs and found that increased intracellular ATP levels improved cancer cell survival (41). A549 NSCLC with increased ATP levels showed increased resistance against sunitinib (54). In addition, cancer cells can internalize extracellular ATP through macropinocytosis and other endocytic mechanisms, contributing to a 1.5-2 times increase in intracellular ATP levels relative to normal cells (54, 56). Drug resistance mechanisms can also result in the enhanced internalization of extracellular ATP. Increased intracellular ATP levels increase the activation of RTKs, preventing the binding of TKIs and inducing RTK-mediated signaling, eventually culminating in drug resistance (41, 54, 56). Extracellular ATP also affects the activity and expression level of the ABC transporter, resulting in the increased efflux of anticancer agents, promoting drug resistance (41, 54). One study reported that ATP levels are associated with purinergic receptor signaling, which promotes cell growth and propagation and contributes to drug resistance (66–68). Cells internalize anticancer drugs primarily through one of three transport mechanisms: 1) passive transfer; 2) facilitated diffusion; and 3) activate the transport (18, 69). Cytotoxic agents can also enter cells using the three ABC transporter molecules in the direction of the concentration gradient; however, drugs internalized into cells using a high concentration gradient typically require active transport (70, 71). Many membrane-localized transporters belong to a family of solute carrier (SLC) transporters. Drug absorption can be reduced either through reduced drug binding affinity or reduced transporter activity. Some chemotherapeutic drugs use specific transporters to enter cells (72), and any mutations in these transporters can inhibit uptake and decrease drug absorption. For example, methotrexate resistance among patients with acute lymphoblastic leukemia (ALL) generally occurs due to gene mutations in human reduced folate carrier (hRFC) (73). A point mutation at nucleotide 133 in the *hRFC* gene results in a lysine to glutamic acid substitution in the first transmembrane domain of hRFC protein that reduces its drug binding affinity. As discussed, both intracellular and extracellular ATP levels play significant roles in the development of cancer drug resistance (73). In addition to the various members of the ABC transporter family, various intrinsic factors, such as p53 loss-of-function, decreased *topoisomerase II* (*Topo-II*) expression, and *bcl-2* oncogene upregulation can promote overall drug resistance (74), as illustrated in **Figure 3**. Moreover, Lung resistance protein (LRP) is found in cytoplasmic vaults and is responsible for the sequestration of anticancer agents into acidic vesicles from the cytoplasm. Although the majority of vaults are found in the cytoplasm, a subset of vaults are found in the nuclear membrane or nuclear pore complex. LRP has the capacity to transfer substrates from the nucleus to the cytoplasm due to the likelihood of LRP localisation in these vaults (74). As a result, the sequestered drugs were unable to cause DNA damage. Additionally, LRP can be used to exocytose anticancer medications from cells *via* acidic vesicles holding the trapped anticancer agents (74). Additionally, LRP and P-gp may be regulated in a similar manner *via* p53 (74). Extracellular ATP can alter ABC transporter expression levels (74), and glucose transporter 1 expression is also related to extracellular ATP levels. Studies have proposed that the involvement of the phosphoinositide 3-kinase–AKT pathway (P2X7-induced) and hypoxia-inducible factor 1α-dependent signaling (53, 66) in the enhancement of cancer cell survival and the development of drug resistance.

## 3 Molecular Mechanisms of Cancer Drug Resistance

**Figure 4:**
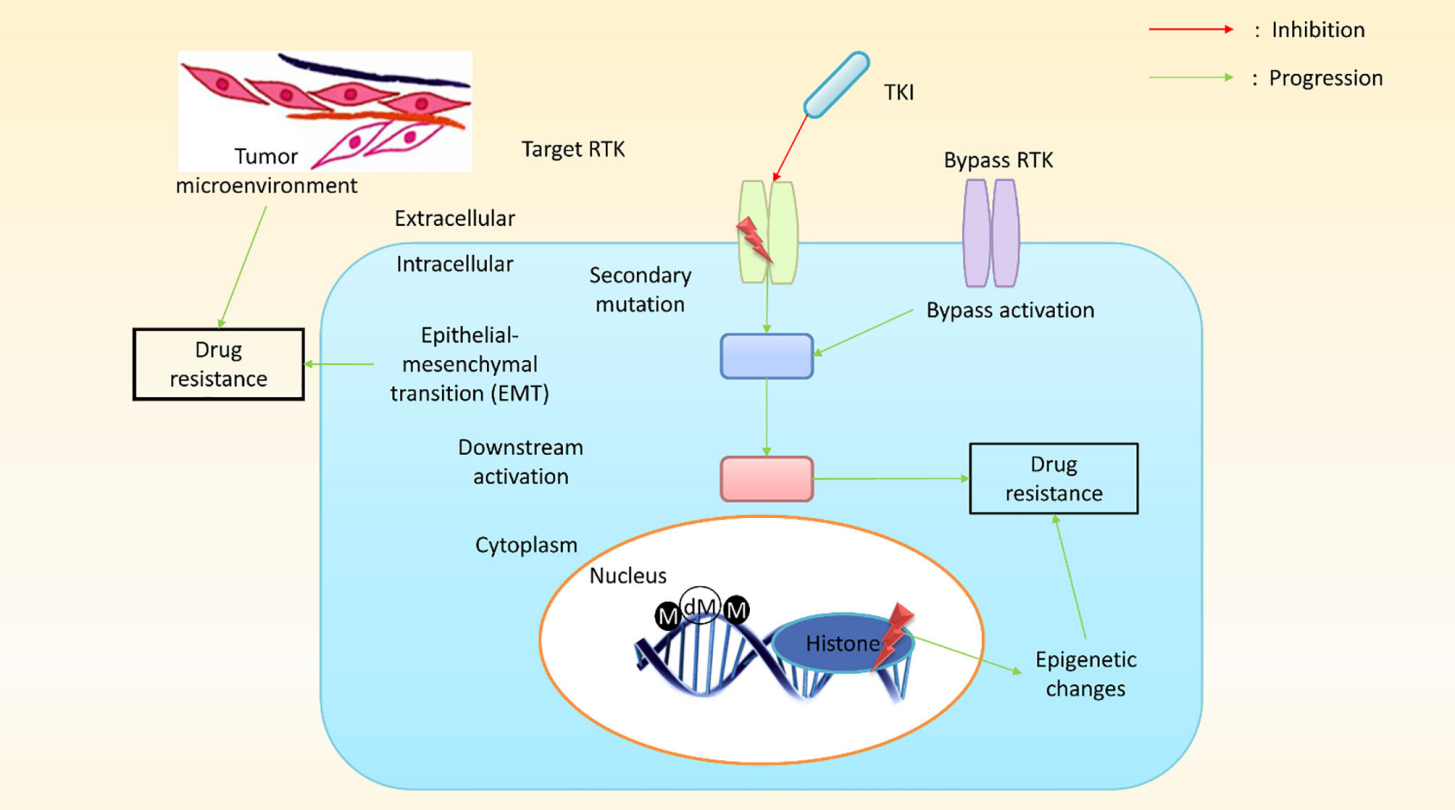
A schematic presentation of pathway-dependent and pathway-independent drug resistance mechanisms in cancer cells. In pathway-dependent (black) mechanisms, a possible target receptor becomes activated, either through overexpression or a secondary mutation (for instance, the kinase domain and ectodomain mutation of epidermal growth factor receptor (*EGFR*) or the overexpression of a truncated version of the target receptor). In addition, gain-of-function mutations in downstream components (e.g., *PIK3CA*, *BRAF*, *KRAS*, etc.) or loss-of-function mutations (*PTEN*, a well-known inhibitor of the downstream pathway) can proliferate downstream pathways. Other possible pathway-dependent molecular mechanisms include bypass activation, leading to the amplification of other isoforms. Pathway-independent (red) mechanisms generally involve epigenetic changes. The epithelial–mesenchymal transition (EMT) in cancer tissues and the tumor microenvironment plays a vital role in developing resistance against cancer treatment. (M, methylation; dM, demethylation; TKI, tyrosine kinase inhibitors; RTK, receptor tyrosine kinase).

Depending on the tissue of origin, the oncogene activation pattern, the activation of tumor suppressors, and differences in gene expression associated with the mutator phenotype of most cancers, cancer cells from a patient can present with widely different genetic backgrounds, and each cancer can express a different array of drug-resistant genes (75). Although cancer cells within a tumor are clonally derived, tumors are characterized by a massive degree of heterogeneity with regard to drug resistance (76). Surprisingly, the primary mechanism underlying MDR in cultured cancer cells is the expression of an energy-dependent drug efflux pump, P-gp, a multidrug transporter (11, 77). In humans, P-gp is the product of the *MDR1* gene (7) and was among the first identified members of the enormous family of ATP-dependent transporters known as the ABC transporter family (72). Research has revealed that *MDR1*/P-gp cannot account for all instances of MDR, suggesting that other drug resistant transporters may also contribute to this phenomenon, such as MRP1 (*ABCC1*) (72, 78) and BCRP (*ABCG2*) (79). In humans, *MDR1* and *MDR2* (a phosphatidylcholine transporter) are expressed in the liver, and defects disrupt the ability to produce bile, resulting in progressive cirrhosis (66–68, 80). P-gp, MRP1, and BCRP are the three most implicated transporters in cancer drug resistance. The molecular mechanisms of cancer cells play pivotal roles in the conceptualization of cancer drug resistance and increasing research has led to an improved understanding of the molecular mechanisms that underlie cancer drug resistance. Research has identified some genetic mechanisms that might result in the development of drug resistance against targeted therapies, which may include secondary mutations, either upstream or downstream of effector activation, and could result in the bypass of certain biological pathways, in addition to epigenetic changes (**Figure 4**).

Molecular alterations in a target protein can also result in acquired drug resistance, such as crizotinib resistance in lung adenocarcinoma, which occurs due to a secondary mutation (G2032R) in the reactive oxygen species (ROS) proto-oncogene 1 (ROS1) kinase domain (75). Crizotinib is a TKI commonly used to treat malignancies associated with anaplastic lymphoma receptor tyrosine kinase (ALK), ROS1, and MET proto-oncogene (*MET*) (81). Similarly, a secondary EGFR mutation in the ectodomain, S492R, results in cetuximab resistance by preventing the EGFR antibody from binding its target site in colon cancer (82). Genetic alterations can also result in signaling protein deregulation, either upstream or downstream of the therapeutic target, resulting in acquired resistance. Research on *EGFR*-mutant cancer cell lines revealed that gefitinib resistance was associated with an oncogenic mutation in phosphatidylinositol-4,5-bisphosphate 3-kinase catalytic subunit alpha (PIK3CA). Moreover, erlotinib resistance can develop due to *EGFR* mutations, as demonstrated in *EGFR*-mutant tumor samples (54, 83). Another proposed mechanism that was recently identified was pathway repetition and oncogenic bypass for targeted anticancer drugs. Secondary RTK activation was reported due to an oncogenic bypass mechanism, resulting in resistance against the primary TKI (84). In addition, EGFR-TKI–resistant squamous lung cancers were associated with the activation of the bone morphogenetic protein signaling pathway (40). Many bypass mechanisms are achieved through feedback loops (85). The examples of immune evasion discussed above represent pathway-dependent mechanisms in which cancer progresses due to the sustained activation of or compensation for a targeted signaling pathway. Pathway-independent mechanisms have also been identified, increasing tumor resistance through EMT, disruption in TME, and angiogenesis moderation. Resistance developed in NSCLC due to the activation of AXL receptor tyrosine kinase (AXL) and the induction of EMT in response to EGFR-targeting anticancer drugs (86). However, another study found that AXL is not necessary for intrinsic resistance maintenance and suggested that the reduced expression of melanocyte inducing transcription factor (MITF) and the overexpression of nuclear factor kappa B (NF-κB) may moderate melanoma resistance to mitogen-activated protein kinase (MAPK) pathway inhibitors (87). Cancer cells can become protected from cytotoxic agents by manipulating the TME, which allows cancer cells to develop acquired resistance, resulting in disease relapse. Studies have shown that the development of innate resistance against RAF kinase inhibitors involves human growth factor (HGF) secretion, which has a considerable impact on the TME (88). The inhibition of BRAF (a TME component) stimulates melanoma-associated fibroblasts, resulting in focal adhesion kinase (FAK)-dependent melanoma survival signaling (89). Epigenetic changes also play pivotal roles in acquired resistance. Studies of epigenetic changes can help define strategies for understanding the limitations of general chemotherapy and targeted therapy. For example, an experiment in PC9 (lung cancer cell lines) cells using an EGFR inhibitor resulted in the development of resistance in sensitive cells, which might be due to a transitional epigenetic state. In addition, the administration of a histone deacetylase inhibitor was able to improve resistance (90). Cisplatin resistance develops in many cancer cells due to DNA methylation, based on the outcomes of DNA methylation and RNA expression profiling (86, 91). Another study found that epigenetic regulators are responsible for the variable responses of different tumors to chemotherapies. Research examining the treatment of solid tumors has explored epigenetic therapies as potential options (92, 93).

## 4 Expression of Resistance Genes

The study of resistance genes can identify the limitations and shortages of cancer immunoprevention strategies. Thus, studying the expression of resistance genes can elucidate the molecular mechanisms that underlay cancer resistance, including their functional roles in cancer cells and normal cells and the conditions under which they are expressed. Much research has focused on understanding the variety of genes that might affect a cancer patient’s samples. By exploring multiple cancer samples, many resistant genes have been identified as oncogenes that amplify the cancer state.

The expression of resistance genes in cancer patients can induce cancer immunoprevention resistance. Multiple genes have been significantly associated with resistance to cancer treatment, as shown in **Table 1**.

**Table 1:** Key molecular function and mechanism of gene expression of resistance genes.

Resistant genes	Sites of expression	Functions	Role	Mechanism	Reference(s)
*ABCB1/MDR1*	Expressed broadly in the adrenal (RPKM=76.0), small intestine (RPKM=43.0) and 8 other tissues	Encodes permeability glycoprotein (P-gp) Involved in the efflux of drugs such as colchicine, taxol, vincristine, daunorubicin, irinotecan etc. It aids in the elimination of xenobiotics and other drug	It functions as a mediator in the development of anticancer drug resistance	Human *ABCB1/MDR1* was isolated from the adrenal and it was found that cDNA, isolated from KB-C2.5, was associated with a Gly-to-Val substitution at position 185, in the predicted cytoplasmic loop between TM2 and TM3.This mutation increased colchicine resistance and decreased vinblastine resistance.	(94, 95)
*ABCC1*	Pervasive expression in testis (RPKM: Reads Per Kilobase of a transcript, per Million mapped reads = 13.5), esophagus (RPKM = 9.9), and 25 other tissues	Encodes multi-drug resistance-associated protein 1 (MRP1). Transportation of drugs means responsible for drug efflux Encodes membrane glycoprotein with 190 kDa and 1531 amino acids	Chemotherapeutic resistance	The promoter region of the *ABCC1* gene carries the AP-1 site, which makes a complex with c-jun/junD. Correlation between the expression of ABC transporters and MAPK may lead to a cancer chemo-resistance pathway	(96, 97)
*ABCC2*	Expressed in the liver (RPKM=24.9), small intestine (RPKM=18.6), and three other tissues	Transport lipophilic substrates with sulfate, glutathione, glucuronate It can regulate the pharmacokinetics of many drugs. *ABCC2* has a function in endogenous metabolites like biliary secretion. Transport variety of xenobiotics	Chemotherapeutic resistance	In TE14 and TE5 cell lines, *ABCC2* appearance was higher and showed powerful resistance to CDDP. ESCC cell lines that contain more *ABCC2* show more resistance to CDDP than lower containment of *ABCC2*. To confirm the role of *ABCC2* in drug resistance, the *ABCC2* gene was silenced with siRNAs into the TE14 cell line raised reactivity to CDDP	(98–100)
*ABCC5*	Expressed in the stomach (RPKM=10.1), spleen (RPKM=6.9, and 24 other tissues	Capable of shifting nucleotide analogs Preparedness for carrying methotrexate Transport exogenous glutamate analogs	Resistance to thiopurine anticancer drugs	Paclitaxel is a chemotherapeutic drug against neck and head cancer. Fork headbox (FOX) molecules are responsible for paclitaxel drug resistance. A molecular study reveals that *ABCC5* with FOXM1 was highly expressed in nasopharyngeal carcinoma cells that were paclitaxel-resistant. *ABCC5* gene transcription is controlled by binding of FOXM1 at the FHK consent pattern of the promoter	(101, 102)
*ABCG2*	Expressed broadly in the kidney (RPKM=44.7), placenta (RPKM=44.0) and 23 other tissues	*ABCG2* encodes breast cancer resistance protein (BCRP) It fuctions as a xenobiotic transporters to exclude xenobiotics from brain Involved in brain-to-blood efflux It plays a vital role in the multidrug resistance phenotype of several cancer cell lines	Involved in resistance to mitoxantrone, daunorubicin and doxorubicin	Increased *ABCG2* expression has been linked to cancer stem cells. The proximal miRNA response element (MRE) of *ABCG2* is located in the 3’-UTR of *ABCG2* mRNA in various cancer cell lines. Interestingly, it was found that this putative MRE of *ABCG2* was lost in drug resistant cells and, therefore, the drug resistant cancer cells can evade *ABCG2* mRNA degradation and protein synthesis repression mediated by miRNAs, leading to over-expression of *ABCG2*	(103–105)
*Bcl-2*	Expressed broadly in the thyroid (RPKM=21.9), spleen (RPKM=9.1), and 20 other tissues	By halting cell death, *Bcl-2* multiplications total cell number. They could modify the shape and energetics of mitochondria At the time of viral infections, *Bcl-2* may modify innate immunity	Involved in resistance to chemotherapeutics and glucocorticoids	*Bcl-2* is an integral part of the mitochondrial and ER membranes. *Bcl-2* is the cardinal pro-survival member that belongs to *Bcl-2* ancestry. *Bcl-2* is capable of binding to the inositol triphosphate receptors, and besides, *Bcl-2* confiscates BH3. Membrane glycoprotein complexes work instead of membrane calcium channels that impaired calcium-mediated apoptosis. Overexposed *Bcl-2* is also responsible for chemotherapy resistance	(106, 107)
*EGFR*	Expressed broadly in the placenta (RPKM=36.6), skin (RPKM=15.6), and 22 other tissues	*EGFR* increases the cell endurance pathway by both kinase-dependent and kinase-independent mechanisms Ligand-operated *EGFR* triggers the proliferation of cells. Ligand Mediated *EGFR* hinders autophagy	Involved in propagating cells	Binding with argonaute two and phosphorylate this protein *via EGFR* results in tumor suppressor miRNAs’ retardation, promoting cancer cell durability. *EGFR* hinders autophagy directly through the phosphorylation of a critical subunit of autophagy initiation complex Beclin-1	(108, 109)
*TP53*	Expressed in the spleen (RPKM=13.2), lymph node (RPKM=13.1), and 25 other tissues	Encodes p53 protein Regulates cell growth Restore DNA damage Control metabolism of cancer Control cell death	Increase resistance to cisplatin, doxorubicin, gemcitabine, tamoxifen and cetuximab	Cancer-deduced p53 mutants are known as *TP53* mutate gene. Approximately among 74% of missense mutations, 80% of them occur in the DNA-binding domain (DBD) of the p53 Two types of p53 mutants are known: DNA-contact mutants and conformational mutants Most of the p53 mutants lose track of native-type function, and some p53 mutants have been obtained GOF (gain-of-functions) that move up chemo-resistance	(110, 111)

## 5 Role of Epigenetics in Cancer Drug Resistance

Epigenetics is the study of heritable phenotypic changes that occur without altering the DNA sequence. Epigenetic remodeling mechanisms have been identified as potential contributors to the development of drug resistance in cancer treatment (30, 41). DNA methylation, histone alterations, chromatin rearrangement, and modifications associated with noncoding RNAs (ncRNAs) are all examples of epigenetic modifications (30, 41, 85). For example, DNA demethylation in an oncogene promoter region activates oncogene expression, leading to the development of resistance (30, 41). According to the study by Ohata et al., a drug-resistant hepatocellular carcinoma (HCC) cell line was associated with an H3 modification in the promoter region. In G-actin monomer binding protein thymosin β4 (Tβ4), it is reinforced DNA methylation (112). *In vivo* study showed that the vascular endothelial growth factor inhibitor sorafenib was ineffective against an HCC cell line due to the excessive expression of Tβ4 (112). Moreover, drug resistance has also been induced in response to chromosome remodeling, ncRNAs, comprised of miRNAs and lncRNAs (113, 114). MiRNAs are tiny ncRNA molecules comprised of 17–25 nucleotides that bind to the periphery of the 3-untranslated region (UTR) of selected mRNAs (115). MiRNAs regulate post-transcriptional gene expression by binding complementary mRNA, causing mRNA degradation and the repression of protein synthesis (18). LncRNAs are also involved in distinct gene expression regulation mechanisms by inhibiting transcription activators that bind to DNA sequences in required genes. Thus, lncRNAs and miRNAs can induce cancer drug resistance by regulating protein expression (41). Histone modifications may alter the chromatin framework (116). Histone acetyltransferases (HATs) mediate histone acetylation events that result in chromatin unwinding, whereas histone deacetylases (HDACs) result in deacetylation events that result in chromatin binding (116, 117). Histone-modifying enzymes and DNA methylation–targeting epigenetic drugs have shown promising results in clinical studies. For example, DNA methylation is inhibited by genistein which is a promising cancer treatment (118). Epigenetic drugs demolish precursor cells in the tumor and reduce cancer recurrence rates (117).

## 6 Regulation of Multidrug Resistance

Despite substantive improvements in anticancer chemotherapy strategies over recent decades, occurrences of MDR have become a great hindrance in the progression of cancer chemotherapies. MDR, which describes the development of resistance to multiple therapeutic agents (119), can develop due to inherent cellular characteristics or be acquired during or after chemotherapy (120, 121). The occurrence of MDR is the product of a sophisticated and multi-factorial process involving a variety of molecular mechanisms. Although no precise biomarkers or underlying mechanisms for MDR have been identified, some principal mechanisms have been identified that are involved in this process, including the overexpression of MDR transporters, defects in the apoptotic machinery, the induction of autophagy, altered drug metabolism, modifications of the drug target, and disruptions in homeostatic redox states (122). Recent studies have suggested that cancer stem cells (123), miRNAs (122), and cytokines (124) play significant regulatory roles in the development of MDR by modulating numerous biological processes. Therefore, cancer stem cells, miRNAs, and cytokines may represent promising biomarkers that can be used to identify and circumvent the development of MDR in cancer chemotherapies.

### 6.1 Multidrug Resistance Regulation by Cancer Stem Cells

Cancer stem cells are a sub-population of cancer cells with the unique abilities to regenerate and differentiate. Cancer stem cells are cancer progenitors and drive the malignancy of many cancer phenotypes, including MDR. A recent study showed that cancer stem cells could be obtained from the human gastric carcinoma cell line SGC-7901 by utilizing the chemotherapy drug vincristine (VCR) (125). This study also suggested that cancer stem cells display mesenchymal properties, including the upregulation of mesenchymal markers and the downregulation of epithelial markers. Matrigel-based differentiation assays showed that cancer stem cells could form tube-like 2-dimensional and lumen-like 3-dimensional structures, resembling the differentiation that occurs in gastric crypts (125). Furthermore, drug sensitivity analyses and cancer xenograft studies indicated that the obtained cancer stem cells display MDR characteristics and remarkable *in vivo* tumorigenicity (125). Another experiment on small cell lung carcinoma demonstrated that the CD133 expression was associated with the development of chemoresistance and increased tumorigenicity in both *in vivo* and *in vitro* studies. The CD133 expression level in cancer stem-like cells were shown to increase in human and mouse models after chemotherapy, which was later substantiated clinically by the longitudinal isolation of specimens from chemotherapy-treated patients. These findings suggest that CD133^+^ cancer stem cells in small cell lung carcinoma display tumorigenicity and chemoresistance properties (126), suggesting a direct relationship between MDR development and cancer stem cells. Existing evidence suggests that cancer stem cells are involved in the mechanism leading to MDR development; therefore, the elimination of cancer or cancer-like stem cells is likely to be necessary to overcome MDR and achieve appreciable prognostication in cancer patients. For example, melatonin and chemotherapeutic drugs have demonstrated synergistically lethal effects against brain cancer stem cells and A-172 glioblastoma cells, associated with the downregulation of ABC transporter expression and function (127). One study of ovarian cancer cells demonstrated that CD44^+^/CD117^+^ stem or stem-like cells have a higher growth rate but a lower differentiation rate after they become resistant to chemotherapeutics (128). Another recent experiment showed that microRNA-199a could significantly increase the chemosensitivity of ovarian cancer stem cells against chemotherapeutic drugs due to reductions in the mRNA expression level of the ABC transporter BCRG (128). In addition, the expression levels of stemness markers were remarkably decreased cancer stem cell lines transfected with microRNA-199a compared with transfection using a microRNA-199a-mutant and untransfected ovarian cell lines. These effects by microRNA-199a are generally attributed to regulatory effects on the target gene CD44 (128).

### 6.2 Multidrug Resistance Regulation by MicroRNAs

MiRNAs are ncRNAs 18–24 bp in length, which modulate the expression of target genes by binding with the 3´-UTR of a target gene (129). MiRNAs play pivotal roles in manifesting lethal phenotypes in cancer cells, including MDR, growth, differentiation, and metastasis among cancer stem cells, and miRNAs can also be used to regulate the abnormal function of target genes (129). For example, miRNA-19a and miRNA-19b, which belong to the miRNA-17/92 cluster, can upregulate MDR in cancer cells and modulate MDR levels in stomach cancer cell lines by targeting phosphatase and tensin homolog (*PTEN*) gene expression (129). MiRNA profiling revealed that miRNA-153 exhibits significantly higher levels of expression in colorectal cancer (CRC) and bowel cancer cells than in normal cells. A recent study of CRC patients over a 50-month period indicated that 21 of 30 patients with increased miRNA-153 levels also displayed increased metastases, whereas lower miRNA-153 levels were associated with reduced metastasis. Furthermore, functional studies demonstrated that increased miRNA-153 levels increased the invasion rate out of CRC cells, and both *in vivo* and *in vitro* studies indicated that they possess resistance against chemotherapeutic cancer drugs, such as oxaliplatin and cisplatin. Moreover, mechanistic studies indicated that miRNA-153 could indirectly promote the cancer cell invasion rate due to the induction of matrix metalloproteinase-9 (MMP-9) enzyme production. However, the direct mediation of drug resistance occurs due to the inhibition of forkhead box (FOX) proteins, especially forkhead box class O 3a (FOXO3a) (130). In addition to cancer-promoting oncomiRNAs, some cancer-suppressive miRNAs have been identified that can induce sensitization in cancer treatments among MDR cancer cells. Studies have shown that the levels of miRNA-15b and miRNA-16, which belong to the miRNA-15/16 family, are decreased in MDR gastric cancer cell line SGC-7901/VCR compared with their expression levels in the parental cancer cell line SGC-7901. *In vitro* drug sensitivity analyses have demonstrated that the overexpression of miRNA-15b or miRNA-16 can sensitize the SGC-7901/VCR cell line against anticancer drugs, whereas the downregulation of these miRNAs using antisense oligonucleotides confers MDR in the SGC-7901 cell line. Furthermore, the overexpression of miRNA-15b or miRNA-16 can induce the sensitization of the SGC-7901/VCR cell line against VCR-induced apoptosis through the regulation of B cell lymphoma 2 (*Bcl-2*) gene expression (131). The overexpression of miRNA-508-5p causes the reversion of cancer cell resistance against several chemotherapeutic drugs *in vitro*, in addition to sensitizing tumor cells against chemotherapeutic agents *in vivo*. In addition, miRNA-508-5p directly targets the 3´-UTR of P-gp and DNA-directed RNA polymerase I subunit RPA12 (ZNRD1) (132). The overexpression of miRNA-27a or the transfection of BEL-7402/5-FU cells with miRNA-27a-like compounds can decrease the P-gp and beta-catenin expression levels and enhance the cellular response to 5-fluorouracil (5-FU), resulting in 5-FU–induced apoptosis. In addition, miRNA-27a upregulation decreased the protein expression of frizzled class receptor 7 (FZD7) without changing the mRNA levels inBEL-7402/5-FU cell lines, and the use of RNA interference to decrease FZD7 protein expression was able to induce miRNA-27a-like inhibitory responses against P-gp and beta-catenin (133). Recent studies demonstrated that miRNAs are involved in the regulation and sensitization of MDR phenotypes and can be utilized as diagnostic markers for MDR occurrence. miRNA-19a levels in serum collected from patients with CRC have been associated with drug resistance, and serum levels of miRNA-19a have complementary values for carcinoembryonic antigen. Further studies have revealed that serum miRNA-19a levels can be used to predict the occurrence of intrinsic and acquired MDR (134).

### 6.3 Cytokines in the Regulation of Multidrug Resistance

The development of effective cancer treatments has been an aim of biomedical sciences over the past few decades (135). Oncoprotein-targeting anticancer drugs represent significant tools in the fight against cancer. Recent studies have demonstrated that distinct cytokines released by cancer-associated stromal cells may result in the development of resistance against chemotherapy-based treatments (136). To better understand the mechanisms underlying cancer drug resistance and predict treatment results, the relationship between cytokines profiles and cancer drug resistance must be established (136). Several cytokines have been used *in vitro* to enhance the cytotoxin sensitivity of MDR cancer cells. The addition of tumor necrosis factor α (TNF-α), interferon γ (IFN-γ), and interleukin-2 (IL-2) to human colon cancer cells resulted in the reduced expressions of the cell lines (137). Sensitivity to chemotherapeutic drugs, such as VCR and DOX, was increased in cells with suppressed P-gp expression, but only if the drugs were administered after P-gp protein expression was inhibited. The study also showed that cytotoxicity does not increase by the subsequent addition of cytokines, which demonstrates that immunotherapy can be used to treat MDR cancers (138). *In vitro* experiments performed in cervical and ovarian carcinoma cells suggested that TNF-α can enhance topo-II inhibitor–mediated cancer cytotoxicity, Also, increased sensitivity of the type II topoisomerases inhibitor was notwithstanding of the TNF-α resistances (139). An *MPR1*-overexpressing breast carcinoma cell line demonstrated inherent sensitivity to *in vitro* cytotoxicity in response to TNF-α (140). Another study showed that IL-2 treatment increased the sensitivity of MDR colon cancer cells to the *in vitro* application of chemotherapeutic agents (141). However, this study has not been substantiated clinically. When designing effective therapies, identifying mechanisms to increase the sensitivity of MDR cancer cell lines to therapeutic agents is necessary. A study of engineered Michigan cancer foundation-7 (MCF-7) breast carcinoma cell lines used to generate xenograft mouse models showed that TNF-α could prevent the *MDR1* gene response against cytotoxic agents. The mouse models containing MCF-7 cell lines together with a cytotoxin-induced TNF-α cassette exhibited a stronger cancer reduction response to DOX-based treatments compared with mouse models containing MCF-7 cell lines overexpressing TNF-α (142).

## 7 Mutational Effects of Drug Resistance

Tumor cells are well-known to become resistant to some chemotherapeutic drugs (143), and many molecular processes contribute to the development of chemoresistance (143). More than 70 oncogenes have been identified that promote cell growth (144, 145). Mutations in these oncogenes affect various molecular mechanisms; oncogenes have been identified that encode membrane growth factor receptors, involved in the growth factor signaling pathway, whereas others encode cytoplasmic signaling molecules. Other oncogenes are involved in the transmission of growth signals, whereas some mutant oncogenes encode nuclear transcription factors, which provide feedback in response to growth signals (143, 144). Recent studies have demonstrated that cell cycle regulators can also act as oncogenes by blocking the apoptotic cell death pathway and promoting uncontrolled cellular proliferation (143, 144). *C-erbB2* encodes an RTK in the EGFR family, which has been characterized as a transmembrane glycoprotein with a molecular weight of 185 kDa (146, 147). Approximately 30% of breast carcinomas patients display *c-erbB2* gene overexpression (148). A clinical research study showed that *erbB2*-overexpressing breast tumor demonstrates reduced sensitivity to methotrexate, cyclophosphamide, and CMF (combination cyclophosphamide, methotrexate, and 5-FU) (149). The detection of *c-erbB2* expression can serve as a chemoresistance marker and predict survival time (150).

Recent studies have indicated the presence of a relationship between signal transduction pathways and chemotherapy responses (143). *Ras*, *v-mos*, *src*, protein kinase C (PKC), and other oncogenes involved in signaling pathways can mediate MDR (151, 152). *Ras* is an oncogene known to be directly involved in human cancer occurrence, with approximately 30% of all human cancers caused by mutations in the *Ras* oncogene (144). The human prostate cancer cell line PC3(R), a variant of PC3 cells featuring HRas overexpression, demonstrated resistance to etoposide, m-amsacrine (m-AMSA), DOX, VCR, and choline phosphotransferase (CPT) (152). Compared with PC3 cells, the levels of P-gp, Topo-I, Topo-II, and glutathione-S-transferase (GST) remain unchanged in PC3(R) cells (152). Thus, the *Ras* gene may be involved in the drug resistance mechanism in PC3(R) cells (143). Genes that depend on activator protein-1 (AP-1) are responsible for cell proliferation, differentiation, tumor cell induction, and chemoresistance (153). Myc class transcription factors may also be involved in the development of chemoresistance (143). *Myc* oncogene-encoded proteins form a sequence-specific DNA-binding complex responsible for DNA repair processes. *L-myc* has been associated with chemoresistance in small cell lung cancer cell lines (154), whereas *N-myc* expression in a neuroblastoma cell increased resistance to cisplatin and etoposide in patients (143). Many chemotherapeutic agents target cell cycle regulators during tumor cell growth (143). The detection of mutations in cell cycle regulators can determine the drug susceptibility of tumor cells (143). Mutations in cyclins and cyclin-dependent kinases (cdks) can affect the cell (143), and a study showed that *cyclin D1* overexpression was associated with drug resistance in a human fibrosarcoma cell line (155). *Cyclin D1* overexpression has been identified in many cancer types, including breast cancer, head and neck cancer, and NSCLC. *Cyclin D1* promotes the progression from the G1 phase to the S phase of the cell cycle, together with cdk4 and cdk6 (156). One study found that mutations in cyclin A, cdk2, and cdk4 increased resistance to staurosporine (157). The study of apoptosis represents an emerging field of cancer treatment. Mutations in apoptotic regulators represent another key factor in the development of MDR (143). Several studies have indicated that bcl-2 protein expression can impaired apoptosis and is involved in the development of MDR (158), and MCF-7 human breast cancer cells with *bcl-2* overexpression are resistant to adriamycin (159).

## 8 Hypoxia-Mediated Drug Resistance

**Figure 5:**
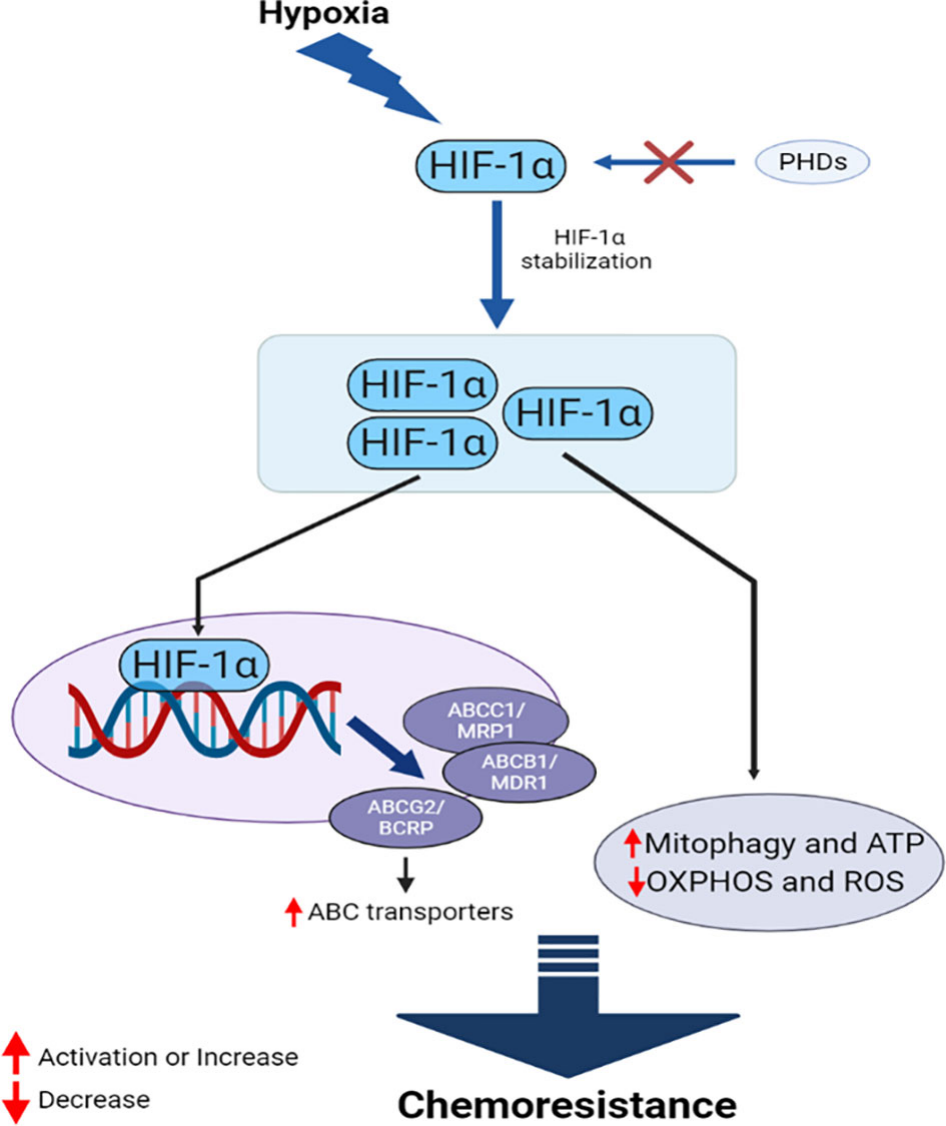
HIF-1α mediates interconnected mechanisms during hypoxia, facilitating chemoresistance in cancer.

Oxygen deprivation in cells and tissues is referred to as hypoxia, and solid tumors commonly exist in a hypoxic state. Cancer cells overcome this condition by either slowing progression, resulting in necrosis/apoptosis, or adapting to the condition. Hypoxia-inducible factors (HIFs) are the primary proteins that allow cancer cells to survive under hypoxic conditions. HIF proteins are dimers consisting of an α subunit, which is generally inactivated by prolyl hydroxylase dioxygenase (PHD) under normal oxygen conditions (160), and a β subunit, which binds to the active subunit under hypoxic conditions, allowing the complex to move into the nucleus freely (161, 162). Three types of HIF-α have been identified in higher organisms, HIF-1α, HIF-2α, HIF-3α, and only one form of HIF-β. HIF-1α mediates the chemoresistance features of cancer cells through multiple and interconnected mechanisms (**Figure 5**). Many chemotherapeutic agents induce cancer cell death by triggering pro-apoptotic pathways, in addition to other programmed cell death pathways, such as necrosis, autophagy, and mitotic catastrophe. The TME demonstrates chemoresistance and limits drug-induced cytotoxicity under hypoxic conditions, promoting malignancy and metastasis. Many anticancer drugs, such as gemcitabine (GEM) (163), DOX, etoposide (164), and cisplatin (165), require oxygen to exert maximal activity, and their functional capacities are reduced under hypoxic conditions. Limited drug bioavailability due to low vascularization is also a feature of hypoxic tumor cells (166). Hypoxia-induced drug resistance has also been associated with the upregulation of oxygen-regulated proteins, the over-replication of DNA, cell cycle arrest, alterations in cellular metabolism, the enhancement of drug efflux pumps, and a lack of genetic stability. The pre-incubation of cancer cells under hypoxic conditions increases resistance to several drugs, as demonstrated in both *in vitro* and *in vivo* studies (12, 13, 167, 168).

HIF proteins are the primary drivers of hypoxia-induced chemoresistance. HIF-1α-targets *MDR1*, which encodes the ABC transporter P-gp (169). P-gp upregulation increases the efflux of anticancer drugs, reducing the intracellular concentration of these drugs and reducing their efficacy. HIF-1α also upregulates the expression of MRP1, BCRP, and LRP under hypoxic conditions (170, 171). Studies have demonstrated that HIF-1α can reduce DNA damage in cancer cells through an unknown mechanism, further contributing to the drug resistance of several cancer types, including triple-negative breast cancer (TNBC) and prostate cancer (PC) (172). HIF-1α also contributes to the DNA repair mechanism and counteracts the activities of several chemotherapeutic agents (173, 174). Furthermore, HIF-1α can increase mitophagy and protect cancer cells from several drugs, including cisplatin (175), 5-FU (176), and GEM (163). Mitophagy also helps cancer cells to replenish ATP, metabolites, and building blocks that have been damaged by drugs. Many anticancer drugs induce oxidative damage as the primary mechanism through which to kill cancer cells, and the reduced capacity for ROS production under hypoxic conditions reduces the efficacy of these drugs (175), likely mediated by the downregulation of oxidative phosphorylation (OXPHOS) by HIF-1α (176). Cancer cells expressing HIF-1α also reduce the pro-apoptotic effects of *TP53* when anticancer drugs, such as cisplatin, are administered (177). HIF-2α mediates a similar phenomenon in hypoxic cancer cells (178). The enhanced upregulation of Pim kinases members, active P-gp, and *Akt*/mammalian target of the rapamycin (mTOR) are also observed under hypoxic conditions, which can induce resistance against several chemotherapeutic drugs, including cisplatin, DOX, and GEM (179–181). HIF-1α can induce resistance in hypoxic cells by increasing the EMT, triggering proliferation and migration in PC (182).

Several miRNAs also contribute to the development of chemoresistance under hypoxic conditions. For example, miR-106a, HIF-1α/miR-210, miR-508-5p, and miRNA-19a/b are known to be involved in the development of chemoresistance (129, 132, 183, 184). Hypoxia-driven autophagy is another protective mechanism activated in cancer cells that can lead to chemoresistance under hypoxic conditions (185–187). The inhibition of ATG5, a mediator of autophagy in hypoxic cancer, can induce cisplatin sensitivity in previously resistant cells (188), supporting the contribution of hypoxia-induced autophagy to the development of anticancer drug resistance. Oxygen deprivation in cancer cells increases acidity due to the increased production of lactate by glycolysis. This acidification of TME can neutralize the activity of drugs that are weak bases (189). Senescence is a key form of programmed cell death associated with many types of stress, such as telomere dysfunction, DNA damage, and oxidative damage. Senescence is more efficient than apoptosis (190, 191). Several anticancer drugs induce senescence to kill cancer cells (192–194), but hypoxia can decrease tumor cell senescence (172). In addition to these intrinsic factors, hypoxia can induce chemoresistance in cancer cells through extrinsic factors. The hypoxic niche in TME can accommodate cancer stem cells, which participate in drug resistance (195). Furthermore, hypoxia-recruited tumor-associated macrophages (TAMs) in the TME release factors that contribute to drug resistance and cancer cell survival (196). Immunogenic cell death mediated by chemotherapy can also be prevented by TAMs in several cancers (197). Some cytokines, such as interleukine-6 (IL-6), are also thought to play roles in hypoxia-induced chemoresistance (198).

## 9 Multidrug Resistance and Autophagy

Autophagy is a conserved cellular process through which damaged or unused proteins, various cytoplasmic elements, or organelles are degraded by moving through the lysosomal system, which allows cells to recycle whole molecules or organelles (199). Autophagy typically involves the formation of a double-membrane body, called the autophagosome, which transports important elements to the lysosome (200). Autophagy is known to play several roles in both cell survival and cell death, acting as a double-edged sword. However, autophagy also plays an important role in the enhancement of chemoresistance in cancer cells. Autophagy can improve the survival of cancer cells during stressful conditions, such as hypoxia, starvation, and damage induced by therapeutic agents (201–203). Autophagy is primarily induced by the inhibition of the mTOR signaling pathway during stress (204) and is regulated by a group of highly conserved genes called autophagy-related genes (ATGs). Studies have demonstrated the contributions of autophagy to the development of drug resistance in cancer cells and have identified various factors associated with this autophagy-induced chemoresistance. The inhibition of autophagy by chloroquine, an antimalarial drug approved by the Food and Drug Administration (FDA), restored the sensitivity to paclitaxel in NSCLC and decreased metastasis by enhancing ROS levels (14, 205). Several ATGs have been identified, including ATG3, ATG5, ATG6, ATG7, and ATG14, which regulate cellular autophagy. An extensive and interconnected ATG network participates in autophagy and mediates drug resistance in cancer cells. Research has shown that ATG3 is directly associated with the development of drug resistance, as the inhibition of ATG3-induced autophagy was able to promote salinomycin-induced apoptosis and enhance the cisplatin sensitivity in NSCLC (206, 207). ATG5 also contributes to DOX resistance, and the upregulation of GBCDRlnc1 (gallbladder cancer drug resistance–associated lncRNA1) in DOX-resistant gallbladder cancer decreases phosphoglycerate kinase 1 (PGK1) degradation and upregulates ATG5 and ATG12 (208). MiRNA-153-3p inhibits ATG5-mediated autophagy and improves sensitivity to gefitinib in NSCLC, further supporting a role for ATG5 in chemoresistance (209). One study showed that ATG5 could also induce macrophage-mediated autophagy in liver cancer, and ATG5 inhibition prevents macrophage-mediated autophagy and restores oxaliplatin sensitivity (15). Another investigation provided evidence that blocking ATG6 (beclin-1) could enhance the efficacy of estrogen receptor (ER)-positive breast cancer cells (210). Other autophagy regulators, such as ATG7 and ATG12, are also involved in chemoresistance. One study demonstrated that the co-administration of silencing ATG7 (siATG7) and docetaxel for breast cancer treatment increased the efficacy of docetaxel-induced apoptosis (211). ATG7 knockout using a small hairpin RNA (shRNA) in AML improved the efficacy of treatment with cytarabine and idarubicin (212). In addition, the shRNA-mediated downregulation of ATG12 resulted in the recurrence of efficacy for trastuzumab, erlotinib, gefitinib, and lapatinib *in vitro* (213). These findings further support the contributions of ATGs to the development of chemoresistance in cancer cells.

Several miRNAs are also involved in autophagy-induced chemoresistance. The relationship between miRNAs (miR-495, miR-30, miR-199a, miR-21, miR-22, miR-410, miR-181, miR-409-3p, miR-26, miR-193, miR-101, and miR-142-3p), autophagy, and chemoresistance has previously been reviewed (214). Among these miRNAs, the downregulation of miR-30a (215), miR-199a-5p (216), miR-410-3p (217), miR-101 (218), and miR-495-3p (219) correlate with drug resistance, including resistance to cisplatin, adriamycin, and GEM, in several cancers through enhanced autophagy. However, some miRNAs, such as miR-21, also participate in drug resistance by inhibiting the protective autophagy of the cell (220). In addition to miRNAs, several lncRNAs are associated with drug resistance induced by autophagy. The roles of various lncRNAs in autophagy-induced chemoresistance have previously been reviewed (6), including bladder cancer-associated transcript 1 (BLACAT1), metastasis-associated lung adenocarcinoma transcript 1 (MALAT1), the X-inactivate specific transcript (XIST), small nucleolar RNA host genes (*SNHGs*), highly upregulated in liver cancer (HULC), and cancer susceptibility candidate 2 (CASC2). These lncRNAs promote drug resistance and stabilize autophagy by downregulating several miRNAs (221–226). However, some drugs can induce autophagic cell death in drug-resistant cancer cells (227). The inhibition of ATG5 can sometimes reduce the efficacy of therapeutic agents, promoting tumor relapse (228). Therefore, the mechanisms through which autophagy affects the development of chemoresistance in cancer cells, are not yet fully understood.

## 10 Laboratory Approaches and Advancements in Cancer Drug Resistance

Currently, most MDR cancer phenotypes can only be identified by separating tumor or cancer cells from primary tissue types and evaluating their tolerance to anticancer or chemotherapeutic drugs due to lack of *in vivo* MDR detection approaches (229). Frequently evaluated cancer drug resistance indicators include the half-maximal inhibitory concentration (IC_50_), half-maximal effective concentration (EC_50_), cell resistance index (RI), the cell growth curve, and the apoptotic index (229, 230). Other assays used to evaluate the drug resistance of cancers and tumor cells include genomic analysis of MDR tumors, drug susceptibility tests in animal models, drug influx and efflux assays, 3-(4,5-dimethylthiazol-2-yl)-2,5-diphenyltetrazolium bromide (MTT) assays, high-content screening and analysis, and high-throughput screening and analysis (231).

### 10.1 Genomic Analysis of Drug-Resistant Cancers

Some genetic factors can confer cancer drug resistance, such as oncogene encoding growth factor receptors (GFRs), cell cycle regulators, signaling molecules, transcription factors, and apoptosis mediators (143). Genomic analysis can be used to identify gene expression to determine the regulatory functions underlying drug resistance in cancer cells (232). Anticancer drug transporter mutations can reduce drug absorption. Patients with ALL harbor mutations in the *hRFC* gene, which confers resistance to methotrexate (18). Mutation at the nucleotide 133 in the *hRFC* gene results in a mutation that prevents drugs from binding to the transporter. Using genomic analysis to assess miRNA expression, DNA methylation, single-nucleotide polymorphisms (SNPs), and single-nucleotide variants, approximately 463 genomic characteristics have been associated with the development of glucocorticoid resistance (232). The discovery of a mutation in a novel gene encoding cadherin EGF LAG seven-pass G-type receptors (CELSRS) results in glucocorticoid resistance, which was identified by network-based transcriptomic modeling and single-cell RNA-sequencing (232). Researchers showed that increased Bcl2 protein expression promoted steroid resistance and reduced the CELSR2 protein level (232). Bcl2 protein impairs the cell death pathway, and the leukemia treatment drug venetoclax inhibits Bcl2 (232). The upregulation of *Bcl2*, *Akt*, and other anti-apoptotic genes, combined with the downregulation of *Bax* and Bcl-xL, which are pro-apoptotic genes, can increase tumor cell resistance to chemotherapy (18). *TP53* gene mutations also impair the functional efficacy of anticancer drugs, in addition to inhibiting the activation of apoptosis. More details regarding cancer drug-resistant genes with their functions can be found in **Table 2**.

**Table 2:** List of some cancer drug-resistant genes with their role and properties.

Drug resistance genes	Role	Properties	References
*ABCB1*	Involvement in multi-drug resistance	➢ Found on chromosome 7 in humans ➢ Size: 1280 amino acids ➢ Molecular mass: 141479 Da	(97, 233)
*ABCC1*	Involvement in multi-drug resistance	➢ Found within the nucleus on chromosome 16 in humans ➢ Molecular mass: 171591 Da ➢ Contained two hydrophobic transmembranes ➢ Size: 1531 amino acids	(97, 234)
*ABCC2*	Involvement in multi-drug resistance	➢ ABCC2 protein belongs to MRP (Multidrug resistance-associated protein) subfamily ➢ Exhibited apical in the part of the hepatocyte ➢ Found on chromosome 10 in humans at position 24.2 ➢ Size: 1545 amino acids ➢ Molecular mass: 174207 Da	(100, 234)
*ABCC3*	Involvement in multi-drug resistance	➢ Belongs to the MRP subfamily ➢ Found within the nucleus on chromosome 17 in humans ➢ Size: 1527 amino acids ➢ Molecular mass: 169343 Da	(234, 235)
*ABCC5*	Involvement in resistance to thiopurine anticancer drugs	➢ Found within the nucleus on chromosome 3 in humans ➢ Size: 1437 amino acids ➢ Molecular mass: 160660 Da	(102, 234)
*ABCG2*	Involvement in multi-drug resistance	➢ Found on chromosome 4 in humans ➢ Size: 655 amino acids ➢ Molecular mass: 72314 Da	(236)
*BCL2L1*	Act as an inhibitor of apoptosis	➢ The formation of BCL2L1 protein is homodimers or heterodimers ➢ Found on chromosome 20 in humans ➢ Size: 233 amino acids ➢ Molecular mass: 26049 Da	(234, 237)
*CLPTM1L*	Elevation cancer susceptibility	➢ Found on chromosome 5 in humans ➢ Size: 538 amino acids ➢ Molecular mass: 62229 Da	(234, 238)
*EGFR*	Increase the propagation of cells	➢ Found on chromosome 7 in humans ➢ Size: 1210 amino acids ➢ Molecular mass: 134277 Da	(234)
*ELK1*	Increase the propagation of cells	➢ Found on the X chromosome in humans ➢ Size: 428 amino acids. ➢ Molecular mass: 44888 Da	(234, 239)
*NFKB1*	Increase the propagation of cells	➢ Found on chromosome 4 in humans ➢ Size: 968 amino acids ➢ Molecular mass: 105356 Da	(234, 240)

### 10.2 Mouse Model Studies and *In vivo* Studies of Cancer Drug Resistance

Studying animal models of chemoresistance due to genetic alterations is vital for the field of cancer biology. *In vivo* models provide a native TME, making *in vivo* studies preferable to *in vitro* studies (241). Animal models must be immunocompromised to prevent the rejection of xenografts consisting of human cancer cells or small segments of chemotherapy-resistant cancer specimens (241). The most commonly used immunocompromised mice for xenograft hosting are (1) severe combined immunodeficiency (SCID) mice, which are B and T cell–immunodeficient mice with defective natural killer cells due to the beige mutation (242); and (2) athymic nude mice (Balb/c, CD-1), which are thymus-deficient mice that fail to produce T cells, and the impairment of T-independent B cell maturation also occurs due to the presence of the *xid* mutation in the nude gene (241). Female 4–5-week-old BALB/c nude mice injected with A549 cells transfected with chromodomain helicase/ATPase DNA-binding protein 1-like gene (CHD1L) shRNA1, shRNA2, or scrambled control shRNA (234) were intraperitoneally treated with cisplatin (3 mg/kg) when tumor sizes reached 5 mm in diameter, which resulted in mouse death, providing evidence that CHD1L exhilarating is responsible for cisplatin resistance (234). Anticancer drug resistance can develop in response to the gene expression of *MDR1* in transgenic mice (243). The anticancer drug, daunomycin, had no effect on a transgenic mouse model expressing human *MDR1* (244). Mice deficient in *mdr1a* and *mdr1b* revealed that P-gp knockout was not fatal in mice but likely increased the assimilation and neurotoxicity of various drugs (10). Knockout mice lacking *mdr1a*/*mdr1b* and *mrp1* genes demonstrated that P-gp and MRP1 transporters were responsible for the development of resistance to anthracyclines, taxol, and vinca alkaloids (245).

### 10.3 Drug Efflux Assays in Cancer Multidrug Resistance

Drug efflux assays are used to test the functional roles of membrane-localized pumps, including P-gp, MRP1, MRP2, and BCRP. Drug efflux assays are performed in living cells under physiological conditions and through direct analysis of the relative fluorescence of cell populations to determine intracellular concentrations of fluorescent MDR probes. Probes utilized in these studies include small-molecule fluorophores and fluorescents for bioimaging, including the classic fluorescent labeling dyes, 3,3´-diethyloxacarbocyanine iodide (DiOC_2_), rhodamine 123 (Rh123), and calcein acetoxymethyl (229). The selection procedure for probes differs across transporters; for example, the evaluation of P-gp is commonly performed using DiOC_2_, Rh123 (246), the antihistamine drug fexofenadine (247), and the cardiac glycoside drug digoxin (248). The primary substance used to assess the BCRP efflux pump is DOX (249), and tariquidar has been as both BCRP and P-gp probes (250). Leukotriene can be transported by MRP1 and MRP2, calcein transported by MRP1, and bilirubin glucuronides are transported by MRP2 (251, 252). A larger group of dyes, including daunorubicin and mitoxantrone, are less sensitive due to their dimness, which can result in false-negative reports (253). The reduction of drug accumulation through enhanced cellular flux is a widely studied mechanism in MDR cancers. ABC transporters are regulatory components found in the plasma membranes of healthy cells, and they mediate efflux. ABC transporters are expressed by humans and other phyla and serve to transport various substrates across the cell membrane. The 49 known ABC transporters are typically comprised of two domains, a highly conserved nucleotide-binding domain and a largely variable transmembrane protein domain (254). An intracellular substrate can be relocated outside of the cell when the hydrolysis of ATP at the nucleotide-binding site causes a change in the conformation, which typically occurs when a substrate binds with the transmembrane protein domain. This efflux mechanism plays a pivotal part in the prevention of toxin over-accumulation in living cells (255). ABC transporters are expressed largely in the epithelial cells of the intestine and liver, where the body expresses proteins to protect against the efflux of drugs and various harmful compounds into the lumens of the intestine and the bile duct. ABC transporters are crucial to the maintenance of the blood–brain barrier (256, 257). Consequently, these three transporters protect cancer cells from various first-line chemotherapies. P-gp was the first identified ABC transporter and has been studied comprehensively (258–260). The expression level of the *MDR1* gene encoding P-gp is typically upregulated in cancerous tissues. However, a study examining both inherent and acquired *MDR1* overexpression mechanisms revealed that DOX treatment might trigger a significant increase in *MDR1* expression levels in lung cancer cells without affecting the expression in normal respiratory cells (261). Lung, prostate gland, and mammary gland tissues do not express *MDR1*, and drug resistance in these tissues is commonly associated with other members of the ABC transporter family, including BCRP and MRP1. BCRP is commonly expressed in stem cells and can protect normal cells from the toxicological effects of xenobiotics by regulating the homeostatic status of heme and folate. Researchers have demonstrated that the upregulation of these transporters in cancer cells can result in worse clinical outcomes, such as the expression of MRP1 in neuroblastoma (262). BCRP expression levels are predictive of drug responses and viability ratios in small cell lung cancer. However, drug efflux can be reduced through the use of BCRP inhibitors, such as gefitinib, a TKI that blocks BCRP transporter function, restoring drug sensitivity (263). Although some compounds have been identified that directly inhibit BCRP, estrogen has also been shown to play a crucial role in the regulation of BCRP expression (79). Cancer cells can be resensitized to the effects of anticancer drug treatment through the inhibition of these transporters.

In addition to activating downstream signaling molecules, kinases are important for maintaining P-gp expression levels and regulating the milieu to develop drug resistance. The translation of P-gp is downregulated by estrogen in estrogen receptor–positive (ER-positive) breast cancer cell lines without affecting estrogen receptor–negative (ER-negative) breast cancer cell lines or DOX-resistant ER-negative ovarian cancer cell lines (264, 265). By contrast, overexpression of proteins in the MAPK pathway results in the activation of downstream tyrosine kinase receptors and the upregulation of P-gp expression. The downregulation of P-gp expression mediated by inhibitory substrates of the MAPK/extracellular signal-regulated kinase (ERK) pathway can be upregulated by some growth factors, such as EGF and fibroblast growth factor (FGF) (266). Furthermore, heat shock protein 90 can stabilize various signal-producing proteins and downregulate P-gp expression. P-gp expression and stability are strictly regulated and necessary for the survival of cancer cells or tumor progression. Targeting cancer-promoting kinase substrates can inhibit P-gp expression by sensitizing cancerous cells to therapeutic drugs.

### 10.4 High-Content Screening of Drug-Resistant Cancers

High-content screening technology can be used to analyze and collect biological data regarding intracellular and intercellular conditions in response to drug stimulation. This system does not require the destruction of cell structures and offers multiple channels for the performance of multiple target detection using fluorescent scanning (267). A high-content screening system is capable of obtaining distinctive cell data, including morphological features, proliferation, cancer differentiation, migration, apoptotic conditions, characteristics of the signal transduction mechanism, and other pertinent information regarding the physiological activity and toxicological effects of various agents within a single experiment (268). A high-content screening assay can be utilized to analyze lysosomotropic substrates, allowing for toxicology screening to identify oncological therapeutics with lysosomotropic properties to be performed using this method (268). This technique revealed the significant contribution of lysosomes to programmed cell death and suggested that lysosome membrane permeability–inducing compounds can be advantageous for the eradication of cancerous cells (269–271).

### 10.5 High-Throughput Screening of Drug-Resistant Cancers

High throughput screening methods generally utilize various molecular and cellular techniques to screen for various outcomes using microplates and are typically applied using automated techniques. High-throughput screening techniques can be utilized to obtain large quantities of data from numerous samples analyzed simultaneously in a single experiment, with accurate and traceable results (229, 269). High-throughput screening and analysis techniques can be utilized to screen functional or phenotypic information, such as the identification of MDR or miRNA gene expression using siRNA or miRNA inhibitory libraries (272). Array-based high-throughput screening methods, including DNA microarrays, cDNA microarrays, RNA immunoprecipitation chips, protein microarrays, protein modification microarrays (such as protein phosphorylation or glycosylation microarrays), can also be utilized for the analysis of MDR gene, RNA, or protein expression (272). For example, by comparing healthy cell lines with drug-resistant cell lines using array-based high-throughput screening, differences in gene expression patterns can be detected, and MDR-associated RNA or protein can be identified (273).

### 10.6 Anticancer Drug Sensitivity Analysis

Drug sensitivity and drug susceptibility tests are performed by analyzing cell proliferation in the presence of chemotherapeutic drugs, which serves as an indirect reflection of cancer cell sensitivity to chemotherapeutic drugs (274, 275). Frequently used techniques at the cellular level are used to measure the growth curve of cancer cells. At the cellular level, anticancer drug sensitivity analysis is commonly performed using three-dimensional microculture techniques, whereas *in vivo* mouse models are used to explore cancer sensitivity at the animal level. The cell growth curve produced through various assays can also be used to examine drug sensitivity at the cellular level (275). The histoculture drug response assay (HDRA) can be used as an organism-level drug sensitivity test through the aseptic removal of cells or tissues from multicellular organisms so that they can function outside of the organism. For example, three-dimensional microcultures utilize a variety of three-dimensional structural cultures to preserve smaller pieces of cancer tissue in glass or plastic culture vessels. The most common culture technique is plasma coagulation, although liquid cultures are becoming widespread. Organic salts, vitamins, amino acids, and serums are used to generate distinctive culture media (275, 276). *In vivo* drug sensitivity can be measured by analyzing the results of chemotherapeutic drugs or molecular substrates on xenograft tumors to evaluate the anticancer or antitumor efficacy of these drugs (277).

Drug sensitivity tests have become very popular, and various types of drug sensitivity tests are used in cancer therapy. Although drug sensitivity tests can be conducted both *in vivo* and *in vitro* (278, 279), the benefits and limitations of *in vivo* and *in vitro* drug sensitivity tests can vary, and each can be adjusted for distinctive clinical circumstances (280). The subrenal capsule assay is one of the most distinguishing and preliminary *in vivo* techniques used to perform drug sensitivity analysis. Tumors from humans are surgically implanted into the renal capsule of a mouse, and anticancer drug sensitivity assays are conducted and analyzed (281, 282). However, orthotopic xenograft models have become more popular of late, in which human cancer or tumor tissues are implanted into immunodeficient mouse models to generate a TME more similar to that observed in humans (280). Because *in vivo* drug sensitivity tests are thought to better simulate the characteristics associated with human cancer proliferation and progression, *in vivo* drug sensitivity tests are viewed as being more clinically relevant than *in vitro* drug sensitivity tests. The use of immunodeficient mouse models is the most recent advancement in the evaluation and prediction of human cancer drug sensitivity, proliferation, and progression (281). By contrast, *in vitro* drug sensitivity testing methodologies are well-diversified and involve the analysis of drug responses and inhibition mechanisms, including chemical, biochemical, cytological, and enzymatic analyses. Some methods for analyzing *in vitro* drug sensitivity include microculture tetrazolium assay (283), ChemoFx assay (284), luminescent ATP detection assay (285, 286), and collagen gel droplet-embedded culture (287). Generally, the efficiency and sensitivity of drugs to affect the enzymatic activity, energy consumption, and cell proliferation can be analyzed by various types of *in vitro* drug sensitivity tests (280). Theoretically, all *in vitro* drug sensitivity analysis techniques share similar biological and pharmacological features. Furthermore, slight changes in the atmosphere do not hamper *in vitro* studies, which can be used to predict the drug sensitivity of cancer or tumor cells. However, a recent study reported that the chemotherapy success rate was associated with the therapeutic efficiency against the clonal or stem cells found in cancer tissues or tumors (288–291). By contrast, *in vivo* drug sensitivity assays do not consistently enhance the outcomes of chemotherapy, specifically in terms of patient survival in clinical practice (292).

## 11 Biomarkers of Cancer Multidrug Resistance

MDR represents a major hindrance to the success of chemotherapy in cancer. Several approaches, including the identification of reliable biomarkers, can minimize the resistance to chemotherapy. Biomarkers refer to molecular changes that can be detected in a biological molecule or system that indicate the presence of unfavorable conditions within the system. The identification of biomarkers is very important for increasing the efficacy of the drugs against MDR cancers. Numerous studies have provided substantial evidence to support the use of several genes, proteins, miRNAs, lncRNAs, and even cancer cell–derived extracellular vesicles (EVs) as biomarkers for predicting drug resistance. Upregulation, downregulation, overexpression, or underexpression of genes, miRNAs, and lncRNA can be used to distinguish between drug-resistant and drug-sensitive cancer cells, enhancing the efficacy of chemotherapy. Proteomic, genetic, epigenetic, and transcriptomic investigations have indicated that specific proteins or genes might serve as potential biomarkers. For example, pancreatic ductal adenocarcinoma (PDAC) is primarily treated with GEM or 5-FU, but some PDAC patients are resistant to GEM and 5-FU. The study demonstrated a negative correlation between SLC28A1 (hCNT1, 606207) and mucin 4 (MUC4, 158372), which serves as a regulatory mechanism that can be used as a biomarker to identify GEM-resistant PDAC patients (293). The expression of the SLC29A1 gene was also identified as a predictive biomarker for GEM resistance but not 5-FU-resistance in a previous study (294). Numerous studies have concluded that SLC29A1 and dihydropyrimidine dehydrogenase (DPYD) might be the most potent biomarkers for optimizing chemotherapy outcomes in PDAC patients (295–297). Another study reported that the overexpression of isocitrate dehydrogenase in resistant glioma cells could also serve as a biomarker (298). In addition, hypoxia induces MDR in several cancers, as described earlier, and hypoxia-related genes, specifically HIFs, could serve as potential biomarkers for identifying hypoxia-induced drug resistance.

The dysregulation of miRNAs in cancer cells can also predict the outcomes of therapy and the resistance to specific drugs, serving as a biomarker. Chen et al. (3) reported that miR-744, miR-574, miR-423, miR-222, miR-140, miR-3178, miR-34a, miR-6780b, and miR-29a contribute to drug resistance in breast cancer. Gasparri et al. (299) reviewed urinary miRNAs in breast cancer and deduced that miR-125b could participate in chemotherapy resistance. Another investigation identified 20 downregulated and four upregulated miRNAs in chemoresistant CRC patients. Among them, six miRNAs, including miR-92a, miR-144-5p let-7i, miR-30e, miR-100, and miR-16, showed consistent dysregulation in later experiments (4). Another experiment identified two new miRNAs, miR-200a and miR-210, that were able to predict drug resistance in metastatic breast cancer when detected at high levels in plasma (300). The overexpression of miR-1229-3p has also been identified in gastric cancer patients who are resistant to 5-FU (301). These reports suggest that these miRNAs may be promising biomarkers of several cancers. In addition to miRNAs, lncRNAs can also be used to predict drug resistance. For example, lncRNA colon cancer‐associated transcript-1 (lncRNA CCAT1) may serve as a potential biomarker for drug resistance in esophageal cancer, as it is expressed abundantly in cancer and has been associated with drug resistance (302). Furthermore, EVs derived from drug-resistant cancer cells may also serve as clinically reliable biomarkers. An overwhelming amount of evidence has indicated their efficiency for use as biomarkers *in vitro* and *in vivo*. However, research reports have confirmed that EVs can transfer the drug-resistant phenotype from drug-resistant cells to drug-sensitive cells in leukemia (303), breast cancer (304, 305), ovarian cancer (306), PC (307), CRC (308), and NSCLC (309). Further, a study also suggested that EVs released from ovarian cancer cells induce cisplatin resistance because they contained DNA methyltransferase 1 (DNMT1) (310). Cancer cells derived EVs also contain several miRNAs and lncRNAs, and studies have extensively reported on the involvement of these miRNAs and lncRNAs in the transmission of drug resistance phenotypes and the extension of drug resistance to several drugs, such as DOX, gefitinib, erlotinib, trastuzumab, and cisplatin (5, 311–316). Based on these results, several clinical trials have evaluated the potential use of EVs as biomarkers for the prediction of MDR development in patients with breast cancer, PC, colon cancer, pancreatic cancer, malignant melanoma, lung cancer, and multiple myeloma, which have been extensively reviewed elsewhere (317).

## 12 Immunoprevention of Multidrug Resistance in Cancer

Prevention is the best method for evading the formidable consequences of cancer, and effective preventive strategies could reduce the global burden of cancer (318). Two well-known prevention methods have been identified in the fight against cancer. One is chemoprevention, and the other one is immunoprevention (318). Chemoprevention may not be effective against MDR cancers due to the resistance mechanisms in cancer cells that allow for the avoidance of drug-induced death during early stages (319). Therefore, Immunoprevention may be the best method for cancer prevention, as early-stage cancer cells may not be able to adopt the necessary evasion measures to become drug-resistant (319, 320). Almost two million new cancer cases were reported in 2020, indicating that cancer prevention is not being applied as a major method in the fight against cancer (1). Cancer Immunoprevention refers to the use of immunological means to stimulate host immune responses to prevent the initiation and development of cancer (25). Immunoprevention is conceptually different from immunotherapy, which induces immunity after tumor onset in patients. By contrast, Immunoprevention aims to eradicate cancer during the early stages by stimulating the patient’s immune system. Host immunity can play an influential role in early tumorigenesis by differentiating between normal cells and tumor cells. The existing Immunoprevention strategies (**Table 3**) focus on vaccines, immunostimulators, and antibodies (25). The concept of cancer Immunoprevention is a relatively new field of research, and very limited data is available to support the ability to completely prevent the development of human cancers (26). However, some studies have highlighted the bright future of this field and the potential to prevent cancer completely. At present, preventive vaccines are the most common goal in the field of cancer immunoprevention. Studies have demonstrated that preventive vaccines are more effective than vaccines targeting antigens during later stages of cancer (343). Two types of vaccines are being explored: vaccines against virally induced cancers and vaccines against non-viral cancers. Hepatitis B virus (HBV) and human papillomavirus (HPV) vaccines are the currently available vaccines that act against virally induced cancer (324, 329, 330). These two vaccines represent the most successful Immunoprevention agents in the field of cancer, which may be able to eradicate drug-resistant cancer before it develops.

**Table 3:** Major immunopreventive agents.

Agents	Preventative outcomes	References
HBV vaccine*	Prevents HBV-induced cancer such as hepatocellular carcinoma (HCC)	(321–324)
HPV vaccine*	Protects against HPV types 16 and 18 and also prevents other HPV-induced cancers such as oropharyngeal, vulvar, cervical, vaginal, penile cancers	(325–330)
HER2 vaccines*	Clinical trials showed reduction in lesions and long term HER2 production in patients with DCIS positive HER2	(331–333)
MUC1 vaccines*	Clinical trials showed strong immune response in patients with intestinal polyps and colon cancer	(334–337)
Immune checkpoint inhibitors	Prevents progression of malignancy of oral premalignant lesions which is showed in preclinical studies	(338–341)
Non-specific immunomodulators (Imiquimod)	Clinical trial showed clearance of actinic keratosis at early stage	(342)

*HBV, hepatitis B virus; HPV, human papillomavirus; HER2, human epidermal growth factor receptor 2; DCIS, ductal carcinoma in situ; MUC1, Mucin 1.

Several studies have demonstrated the effectiveness of vaccines against non-viral cancers. Based on the results of preclinical studies, some of these vaccines have entered clinical trials with promising efficacy. Although the use of these vaccines may face limitations due to the occurrence of side effects, they also illuminate the path of future Immunoprevention strategies for cancers. For non-viral vaccine targets, studies have suggested numerous potential antigen targets in several cancers (344). Among these antigens, mucin 1 (*MUC1*) represents one of the most promising targets for the development of protective vaccines, as the overexpression of this gene is associated with colon, pancreatic, breast, and various other carcinomas (345). A peptide-based vaccine that targets MUC1 antigen is in development and currently enrolling patients in clinical trials (337). Mutated *HER2* is another oncogene that is being targeted for preventive vaccines in several studies (346), as HER2 is a promising target for the prevention of breast cancer. Some patients with ductal carcinoma *in situ* (DCIS) who received the HER2 vaccine showed reductions in lesion size in clinical trials (332). In another trial, patients with *HER2*-overexpressing DCIS received dendritic cells displaying HER2, and 25% of them demonstrated complete tumor regression (333). The anti-EGFR vaccine resulted in a 76.4% reduction in EGFR-induced lung cancer in mice (347). Several epitopes, such as syndecan-1 (CD138), X-box–binding protein 1 (XBP1)-unspliced, XBP1-spliced, CS1, have been identified as potential targets in multiple myeloma (MM) and smoldering multiple myeloma (SMM), and their applications against MM and SMM were also very effective (348–350).

However, vaccine-mediated approaches are not successful in every individual, as vaccines mediate their effects through the stimulation of the recipient’s immune system. Many individuals have suppressed immune systems, which is a major contributor to the failure of vaccines and other immunotherapies (351). Several factors contribute to immune suppression, and the inhibition of these factors may represent one potential pathway to overcoming the limitations associated with cancer immunoprevention. The polyfunctional myeloid-derived suppressor cells (MDSCs) are an important inducer of immune suppression (352–354) that is often detected in early lesions during tumor development (355, 356). The first preventive vaccine was tested in individuals with a recent history of adenoma of the colon, and 43% of patients showed a response to this vaccine; however, patients with high levels of MDSC failed to respond to the vaccine. These reports suggested that the inhibition of MDSC may serve to enhance the efficacy of Immunoprevention methods in cancer, which has been supported by the findings of several studies (357–360). Other factors, such as the upregulation of immune checkpoint molecules, including programmed death-ligand 1 (PD-L1) and programmed cell death protein 1 (PD-1), also suppress the immune system (361). Ongoing Immunoprevention trials, including NCT03692325, NCT03347838, and NCT03603223, are exploring the safety and efficacy of using nivolumab, pembrolizumab, and anti-PD-1 molecules, respectively. However, preclinical studies have demonstrated that PD-1 blockade can completely reduce the progression of oral premalignant lesions (362). Other approaches have been explored to prevent cancer during the early stages. Several non-specific immuno-modulators have been explored as potential Immunoprevention agents in cancer. The use of lenalidomide resulted in a 70% risk reduction in clinical malignancy (363). Imiquimod is an approved cream used to treat actinic keratosis (AK) that has demonstrated preventive properties in a clinical trial and protected patients with a history of long exposure to sunlight but without AK. Skin inflammation and AK were diminished after treatment with 5% imiquimod for eight weeks (342). The findings of these studies suggest that Immunoprevention could be a useful prerequisite to treat MDR in cancer cells (138). Although many efforts and various approaches have been used, the optimal immunological strategy for the treatment of MDR cancer cells remains uncertain. With incremental progress, Immunoprevention methods may eventually be combined with other agents to impede tumor cell proliferation (138). The use of various approaches to the eventual goal of cancer Immunoprevention may eventually lead to useful, practical, and improved methods (25).

### 12.1 Limitations and Future Perspectives for Immunoprevention

Despite many aspects, efforts, and approaches to the goal of developing a cancer Immunoprevention method, potential side effects, toxicities, mutations, and immune checkpoint modulations remain significant concerns and restrictions that impede this method from reaching clinical application (25). Scientists must scrutinize the mechanisms of reaction, which may not effectively target cells that do not present the target antigens and are less vulnerable to autoimmunity (364). The activation of specific antigen-presenting cells may not be possible, and the development of personalized vaccines may be necessary (365, 366). In addition, major histocompatibility complex (MHC) glycoprotein polymorphisms and the peptide presentation range represent a significant limitation (364). Thus, the researchers must pay greater attention to the effects played by the mode of administration and the possible side effects of various drugs and vaccination programs to facilitate the successful development of Immunoprevention methods (25). In recent decades, cancer immunology has become a field with growing interest (367). Immuno-preventive actions should be combined with the large-scale screening of cancer prophylactics (368). Advancements in the field of cancer Immunoprevention are regularly being achieved (367). Recently, three types of HPV preventive vaccines have been successfully administered to cervical cancer risk groups based on Pap test results (369). Although various obstacles exist that make the development of immuno-preventive methods challenging, progress has been made in various areas, including the improvement of targeted cancer immunotherapies, optimizing treatments by providing cancer immunotherapy in combination with drug treatments, and Immunoprevention strategies. Critical innovation pathways and new research prospects will eventually lead to the overcoming of these obstacles (367).

## 13 Alternative Therapeutic Approaches against Multidrug Resistance

Cancer cells can avoid cell death by preventing drug-induced cytotoxicity, apoptosis, and autophagy, becoming resistant against drugs using previously described methods. Researchers have explored various methods and avenues to overcome drug resistance. Microparticles, nanomedicines, and gene editing techniques, such as CRISPR/Cas9, are among the modern-day tools that are currently being explored for use against cancer, especially MDR cancer. Many of these methods have shown tremendous results in preclinical studies and are currently undergoing several clinical studies. Despite their promising preclinical outcomes, very few have obtained approval for public use. Here, we have extensively reviewed some of these techniques, which have been tested against drug-resistant cancers.

### 13.1 Microparticles in the Prevention of Drug Resistance

Microparticles (MPs) are enveloped plasma membrane fragments that are also sometimes classified as microvesicles (MVs), with an average size between 100 and 1000 nm. MPs are thought to be released from cells during apoptosis or cellular activation due to the loss of phospholipid asymmetry when phosphatidylserine is relocated from the inner side of the plasma membrane to the outer side (370). However, tumor cells also continuously shed MPs from their surfaces, and evidence suggests that tumor cell–derived MPs are involved in the development of MDR, transferring functional resistance proteins from donor-resistant cells to drug-responsive cells in as little as several hours (303, 371, 372). MPs might also act to sequester anticancer drugs by expressing the drug efflux transporter P-gp on their surfaces, as an active mechanism, and through the diffusion of chemotherapeutic drugs, such as DOX, anthracyclines, and daunorubicin across the MP membrane, as a passive mechanism (120, 373). Studies have also reported their participation in MDR by inducing the metastatic capacity of several cancers (374–377). These reports indicate that cancer cell–derived MPs could represent promising therapeutic targets for the prevention of MDR in cancer. The inhibition of MPs may provide an alternative mechanism for reversing MDR in cancer. The study of MPs is growing into a promising field, and several approaches have been examined for the blockade or modulation of MP production by cancer cells. Several studies have reported the successful inhibition of MPs using various types of inhibitors, which can be further considered for application to the prevention of MDR in cancer. Calpains are important for MP formation, and calpain expression has been identified in several cancers. Calpain inhibition can abolish MDR (378, 379), and calpain inhibitors, such as MDL-28170 (380) and calpeptin (381), can reduce MPs and enhance the sensitivity to trastuzumab in breast cancer (382). Moreover, Rho-A, Rac, cell division control protein 42 homolog (Cdc42), LIM kinase (LIMK), and Rho-associated protein kinase (ROCK) are also important for MP biogenesis. The blockade of Rho-A expression by adenovirus-induced RNA interference decreased MP synthesis in cervical carcinoma HeLa cells (383). Another inhibitor, AZA1, successfully inhibited both Rac1 and Cdc42, resulting in reducing cell migration and growth in PC (384). ROCK inhibitors, such as AT13148, also showed suppressive effects toward metastasis, and MDR and AT13148 entered phase I clinical trials for solid tumors (385, 386). Other MP inhibitors, such as eicosapentaenoic acid/docosahexaenoic acid, BPTES, 968 (Bromo-di-benzophenanthridine), ticlopidine, clopidogrel, LMP- 20, statins, pantethine, and cystamine, have also been found to lower MP levels and have been used for the immunoprevention of MDR in cancer (387–390). Although these drugs lower MP levels, they fail to reduce MP levels to those observed in health controls; therefore, more research remains necessary to identify appropriate inhibitors or modulators that are effective against MP production (391, 392).

In addition to the negative contributions of MPs to MDR development, their capacity to carry a variety of components can be exploited in drug delivery. Modified or artificial MPs can be used as drug delivery systems for better chemotherapeutic outcomes in MDR cancer cells. Two primary challenges can prevent the reversal of drug resistance: one is the insufficient contact of cancer cells with anticancer drugs, and the other is the insufficient internalization of drugs into targeted cells. MP-encapsulated drugs can be used to overcome these challenges (393, 394). Clinical observations revealed that chemotherapy could result in adverse events, including cardiotoxicity, organ damage, and myelosuppression which also represent major obstacles to the reversal of drug resistance through the application of small molecules to block MDR genes (395, 396). However, MP-encapsulated drugs showed improved drug delivery, resulting in decreased systemic toxicity and organ damage (393, 397). These MPs can provide unique advantages to drug delivery, such as better safety and improved cellular affinity, enhanced physiochemical features of drugs, and prevention of inappropriate distribution to normal tissues, reducing organ damage and cytotoxicity and resulting in better uptake by tumor cells to increase drug aggregation (393, 394). A study showed that an MP-packaged survivin inhibitor, YM155, applied to MDR osteosarcoma displayed increased anticancer capacity with reduced organ damage and systemic toxicity (398). Another study demonstrated the improved uptake of drugs in cancer cells and showed better results when the researchers modified a human mesothelin antibody with acid-prepared mesoporous spheres, non-toxic, amorphous, silica microparticles, compared with control in chemoresistant malignant mesotheliomas (399). Although MPs can contribute to MDR development through several mechanisms, these reports confirm that modified MPs can also be used to reverse MDR as drug carriers.

### 13.2 Nanomedicine-Based Approaches

**Figure 6:**
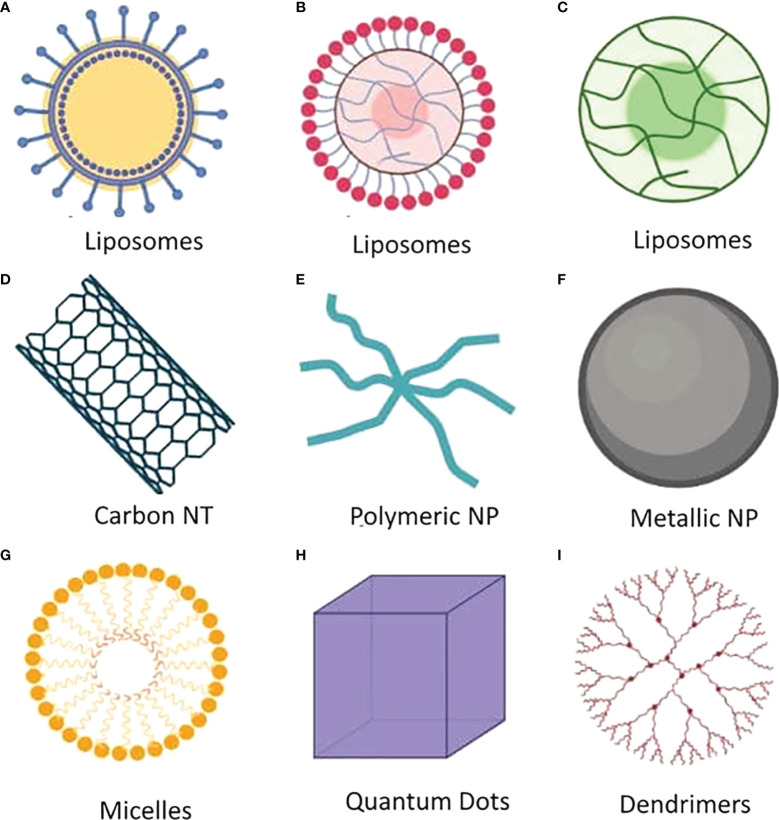
Notable nanoparticles that have several applications in the field of medical sciences. **(A)** Multilamellar liposomes which have several phospholipid bilayer spheres. **(B)** Large unilamellar liposomes which have single phospholipid bilayer sphere and size of 200 to 800 nm. **(C)** Small unilamellar liposomes which also have single phospholipid bilayer sphere and size of less than 100 nm. **(D)** Carbon nanotubes are made of sheets of single-layer carbon atoms. **(E)** Polymeric nanoparticles which have size ranging from 1 to 1000 nm and also known as colloidal solid particles. **(F)** Metallic nanoparticles are made of metal as core and organic compound or inorganic metal as sphere. **(G)** Micelles are composed of amphiphilic macromolecules which range from 5 to 100 nm as nanoparticle. **(H)** Quantum dots are ultrasmall semiconductor nanoparticle. **(I)** Dendrimers are nanoparticle organized with core, inner shell and outer shell.

For the past few decades, nanomedicine has been exploited to overcome the MDR in cancer (400). Nanomedicine refers to nanotechnology-based medicine that takes advantage of the physiochemical features of nanomaterials to develop medical applications (401). The level of interest in nanomedicine in cancer is increasing, and several nanomedicine-based approaches have been explored to address MDR in cancer (402). Nanomaterials, such as nanoparticles (NPs), have demonstrated great efficacy for the treatment of MDR cancer, providing biodistribution control, efficient drug release in resistant cancer cells, and promising storage capacity, which can transform low therapeutic indexed drugs into promising ones (400, 403). NPs have demonstrated the best results when they are used as a delivery system for anticancer drugs. Drug delivery using NPs encapsulation results in increased half-life of drugs and better accumulation in the tumor (404). Several classes of nanoparticles (**Figure 6** **)** have been developed, including liposomes, micelles, polymeric nanoparticles, dendrites, and inorganic nanoparticles, and explored for applications in overcoming MDR. NPs have previously been well-reviewed (405). Some NPs have already obtained FDA approval and are in the process of being developed for clinical use based on their capacity to reverse drug resistance in preclinical studies (28).

Liposomes have been extensively studied for the treatment of MDR cancer. Liposomes are spheres of fatty acids with remarkable features, such as the capacity for self-assembly, easy characterization, and biocompatibility. Several experiments have been performed to verify their efficacy, and based on these experiments, liposomes received FDA approval as the first nanomedical drug delivery system (406). Liposomes have also demonstrated efficacy in the treatment of MDR cancer. A study showed that nanomedicines constructed using lonidamine liposomes and epirubicin liposomes were able to reverse MDR development in NSCLC and achieve enhanced treatment efficacy (407). Liposomes containing DOX showed better drug release and enhanced cytotoxicity in VCR-resistant human leukemia cell lines (408) and in drug-resistant brain tumors in rats (409). Vidhi et al. built a liposome delivery system for vinblastine (CPD100), which was able to prolong the drug circulation time and suppress tumor growth during hypoxia without any adverse effects (410). Scientists have also developed liposomes combined with an anti-EGFR aptamer (Apt) to target *EGFR* muted cancer cells using erlotinib (411) and later observed improvements in the reversal of hypoxia-induced resistance when they applied perfluorooctylbromide (PFOB) using the liposomal formulation (412). In addition, the co-administration of DOX and siRNA using liposomes to treat MDR tumors exhibited better DOX uptake by avoiding the P-gp-mediated efflux effect (413). The application irinotecan-releasing benzoporphyrin and nitric oxide-releasing DOX loaded into a liposome-like nanoformulation was able to respectively reduce *ABCG2*-mediated resistance and MRP1- and P-gp-mediated resistance (414, 415). An *in vivo* study also demonstrated the efficacy of epirubicin and antisense oligonucleotides against P-gp, MRP1, and MRP2 using loaded pegylated liposomes in a CRC model (416).

Inorganic NPs, such as silver (Ag), zinc oxide (ZnO), iron oxide, and gold (Au), are often functionalized against drug-resistant cancer. Au is the most commonly utilized inorganic NP. DOX encapsulated in Au nanoparticles showed higher uptake and induced greater cytotoxicity in drug-resistant cells, such as breast cancer cells (417, 418), in addition to the better accumulation of drugs in tumor tissues (419). Au NPs also decreased GEM resistance in cancer (420). In addition to Au NPs, silver NPs have been explored in various investigations and were found to cause the downregulation of *Bcl-2* and Bcl-xL other similar anti-apoptotic genes and upregulate *Bax*, *Bad*, and *Bak*, which are pro-apoptotic genes, in CRC and lung cancer (421–425), which could contribute to overcoming MDR. Copper cysteamine NPs and fluorescent nanodiamonds have also been found to be effective for inducing apoptosis, tumor suppression, and overcoming MDR (426, 427). Polymeric nanoparticles have demonstrated impactful effects in reducing drug resistance. Polymeric structures have been designed to associate efflux inhibitors and to carry drugs and nucleic acids more efficiently, resulting in enhanced drug accumulation in drug-resistant tumor cells. Several investigations have demonstrated their usefulness in improving MDR. A study showed that the co-administration of paclitaxel and survivin-targeted shRNA NPs improved the efficacy of paclitaxel in paclitaxel-resistant lung cancer, whereas the application of polymeric NPs loaded with paclitaxel showed better target specificity and decreased adverse effects (428). Scientists constructed a system named miR-200c-loaded PEEP-PEDP polymersome, featuring a combination of polyphosphazene, [NP(PEG)0.5(DPA)1.5]n (PEDP), and amphiphilic [NP (PEG)0.3(EAB)1.7]n (PEEP) carrying miR-200c. This nanosystem was demonstrated to induce antitumor activities in paclitaxel-resistant cancer cells (429). The administration of polymeric NPs and ceramide-encapsulated paclitaxel has been reported to circumvention MDR in human ovarian cancer cells (430). Other NPs, such as micelles and dendrites, have also been explored for their usefulness against drug resistance. Micelles are known for their drug-carrying capacity and the ability to escape from drug efflux in resistant cells. A study reported that paclitaxel coated with micelles was able to escape the efflux mechanism, increasing drug uptake in drug-resistant tumor cells (431). Micelles loaded with DOX and methotrexate can also overcome MDR and prevent tumorigenesis (431, 432). Another study demonstrated that antibodies against structural maintenance of chromosomes protein 2 (SMC2) and 5-FU loaded into micelles could overcome resistance to 5-FU in human CRC lines (433). Recently, several anticancer drugs loaded in micelles have entered into clinical trials (434). In addition, the combination of dendrimer and DOX was found to exhibit high cytotoxicity against both drug-resistant cancer cells and drug-sensitive cells (435, 436). DOX coated with dendritic mesoporous silica NPs was also found to be effective against CRC (437).

Other approaches are being explored using different types of nanomaterials. The administration of curcumin with DOX in a copolymer vehicle was found to increase drug accumulation and decrease tumor cell migration (438). Similarly, VCR in NPs resulted in increased drug accumulation and cytotoxicity in drug-resistant cells (439). DOX encapsulated in poly (lactic-co-glycolic acid) (PLGA) demonstrated a similar result and reversed MDR in drug-resistant human breast cancer (440). The application of DOX in carbon nanotubes resulted in better DOX release in resistant human leukemia cells (441). The administration of an ultrasound activatable nanomedicine composed of ferrate and DOX loaded in mesoporous nanoplatforms with *n*-heneicosane demonstrated the ability to overcome hypoxia-induced resistance by downregulating HIF-1α and MDR expression (442). Nanoformulations, such as paclitaxel in nanocrystals (443), DOX in poly (aspartic acid) NPs (444), paclitaxel with phosphatidylserine lipid nanovesicles (445), and DOX loaded in magnetic silk fibroin-based NPs (446), also demonstrated efficacy against MDR cancer in several studies. DNA damage repair is one mechanism for drug resistance. Poly (ADP-ribose) polymerase 1 (PARP1) participates in DNA damage repair in cancer cells. Nanoparticles can be used to effect efficient PARP inhibition in several cancers (447–449).

### 13.3 Applications of CRISPR/Cas9 Technology

In the past few years, CRISPR/Cas9 has been tested for the treatment of cancer and cancer-related complications. Numerous investigations have demonstrated that this genome editing technology can disrupt and modify genes in a variety of cancer cells, resulting in cancer cell death (450, 451). CRISPR/Cas9 and its applications in cancer treatment are likely to represent a significant game-changer for overcoming MDR features in cancer. Although this technology has not yet reached human clinical trials, several preclinical studies have demonstrated the efficiency of this system to reverse MDR in cancer. The CRISPR system utilizes an endonuclease enzyme named Cas9 combined with a single guided-RNA (sgRNA), and the components of the CRISPR/Cas9 system can be delivered using various delivery systems, such as viral vectors, plasmid DNA, NPs, or cationic polymers (452–454). Several studies have suggested that inhibiting resistance factor(s) in cancer cells can represent an attractive method for preventing the development of drug resistance. Researchers used CRISPR/Cas9 to delete genes that can induce resistance to anticancer drugs and achieve successful outcomes in both *in vitro* and *in vivo* studies. Ha et al. applied the CRISPR/Cas9 system to the *MDR1* gene in drug-resistant breast cancer MCF-7/ADR cells and found that the sensitivity of DOX was increased using this method (455). *ABCB1*, another drug resistance–inducing gene, was knocked down using CRISPR/Cas9 in one study, resulting in enhanced drug sensitivity in adriamycin-resistant ovarian cancer cell lines (29). The results from this study also suggested that CRISPR/Cas9 could successfully decrease the expression of P-gp. Further, Wang et al. reported reduced resistance to cisplatin, docetaxel, DOX, and 5-FU in drug-resistant cancer cell lines following the application of CRISPR/Cas9 to knock out urokinase plasminogen activator receptor (uPAR), which is overexpressed in several cancers (456). CD44 is a cancer cell marker that is abundantly expressed in many types of cancers. The expression of CD44 is higher in drug-resistant cancer cells than in drug-sensitive cancer cells. CRISPR/Cas9 can silence CD44, resulting in a decrease in the expression of P-gp and the restoration of drug sensitivity in drug-resistant osteosarcoma (457). Terai et al. also exploited a genome-wide CRISPR system with an EGFR-TKI in *EGFR*-mutated lung cancer cell lines (458). CRISPR-mediated knockout is also able to reduce drug resistance in lung cancer cells and HCC cells (459, 460). The CRISPR-induced repurposing of drug resistance–mediating lncRNA was also able to restore drug sensitivity in cancer cells (461).

CRISPR-mediated screening and identification of drug resistance genes could be conducive to the redesign of therapeutic strategies against drug-resistant cancer. The identification and screening of drug resistance using CRISPR/Cas9 demonstrate high reagent consistency and high validation rates (462). Several mutated gain-of-function and loss-of-function genes play critical roles in drug resistance, and their identification is crucial to overcoming MDR in cancer. In one study, some researchers used a CRISPR/Cas9 knockout screen to identify 10 drug-resistant genes, including C1orf115, a previously uncharacterized MDR gene that they named required for drug-induced death 1 (RDD1) (463). Studies also uncovered other information using CRISPR-based systems, which could further promote research toward overcoming drug resistance. A genome-wide CRISPR/Cas9 screening found that the loss of Kelch-like ECH-associated protein 1 (KEAP1) (464) and SGOL1 (465) promote drug resistance in cancer cells, and the CRISPR-induced deletion of *SLFN11* (466), *BAK* (467), and any CST complex members (468) can initiate drug resistance in cancer cells. However, CRISPR/Cas9 has some limitations, including potential immunological risk. Another concern in employing CRISPR/Cas9 in clinical trials is low efficiency due to low specificity and the safety of delivery into cells (469). However, carefully designed CRISPR systems would be game-changing against MDR in cancer, particularly in the current era of personalized medicine.

## 14 Risk Factors and Possible Threats of Drug Resistance in Cancer Treatment

In cancer treatment, the risk factors associated with particular cancer types should always be considered. Determining the exact causes that drive cancer development can be difficult, and several risk factors may strengthen cancer. As discussed above, exploring the molecular mechanisms underlying drug resistance by performing genomic analyses, identifying the expression of resistance genes, and identifying epigenetic alterations, can improve understanding of the drivers of drug resistance, which can include various risk factors in the history of cancer patients. Risk factors can have significant effects on cancer development, progression, and resistance to cancer treatments. However, risk factors vary from patient to patient and differ among the many known cancer types. Some common risk factors are briefly described in **Table 4**.

**Table 4:** Risk factors of drug resistance in cancer treatment.

Some types of cancer	Risk factors	References
Pancreatic cancer	➢ Family history ➢ Smoking of tobacco ➢ Chronic pancreatitis ➢ Diabetes ➢ Obesity ➢ Hazard experienced in the workplace	(470)
Endometriosis associated ovarian cancer	➢ Lack of the removal of lymph nodes in the tumor area ➢ Positive lymph nodes ➢ The previous report of breast cancer	(471)
Epithelial ovarian cancer	➢ Overexerted Hexokinase II ➢ Duration of the menstrual cycle ➢ Replacement therapy of estrogen ➢ Family history	(472, 473)
Lung cancer	➢ Tuberculosis infection in family history ➢ Tobacco smoking ➢ Tumor in family history ➢ Briny fish	(474)
Colon cancer	➢ Adenomatous Polyps in family history ➢ Ulcerative colitis and Crohn disease history ➢ Animal fat in the diet ➢ Tobacco smoking ➢ Alcohol intake	(475)

Anticancer drug resistance is problematic during cancer treatment, associated with over 90% of all cancer patient deaths (476). Drug resistance in cancer treatment results in 1) the failure of standard treatment, 2) long-term illness, 3) expensive health care costs, and 4) increased mortality (477).

## 15 Conclusions

The effectiveness of chemotherapy is significantly limited by the expression of MDR genes, which play key molecular and cellular roles in the induction of resistance against anticancer agents. Drug resistance can occur due to genetically unstable human cancer cells, and drug-resistant features can either present before treatment (intrinsic) or develop after therapy (acquired). Drug resistance is responsible for most cancer relapses. Anticancer agents can fail to inhibit cancer cells and tumor suppressor genes for various reasons, including mutations in genes and gene amplification, epigenetic alterations, drug efflux enhancement, apoptosis suppression, and alterations in drug metabolism. MDR genes, such as *MDR1* and *MDR2*, are highly responsible for anticancer resistance, combined with various extracellular and intracellular factors. They increase epigenetic gene expression, EMT, and anti-apoptotic factors and significantly decrease P53 tumor antigens. The most common drug resistance mechanism is the efflux of hydrophobic drugs, mediated by ATP-dependent ABC transporters, such as P-gp, an integral membrane protein that is overexpressed in various malignancies. The broad substrate specificity and abundance of ABC transporter proteins represent significant challenges to circumventing ABC-mediated MDR *in vivo*. However, several new drugs, small molecules, and monoclonal antibodies have been identified that directly target oncogenic factors. Currently, treatments with immune checkpoint inhibitors and vaccines represent the most effective management techniques for cancer. We summarized the functional roles of drug-resistant genes and other crucial factors that contribute to drug resistance mechanisms in cancer and highlighted cancer Immunoprevention and other approaches to combat drug resistance. We also elucidated other mechanisms that can be used to better understand drug resistance mechanisms, providing guidance for future cancer treatment to achieve better outcomes.

## Author Contributions

Conceptualization, TE and AS; data curation, TE and AS; writing—original draft preparation, TE, AS, AM, TR, MA, MF-RS, HA, and NR; validation, TE, AS, and MF-RS; formal analysis, TE, AS, and MF-RS; investigation, TE, AS, AM, TR, MA, MF-RS, HA, and NR; resources, TE and MoMH; writing—reviewing and editing, TE, AS, AM, MF-RS, HA, FN, EW, SM, KD, MaMH, SH, AI, and MoMH ; visualization, TE, AS, AM, MF-R-S, HA, FN, EW, SM, KD, MaMH , SH, AI, and MoMH ; supervision, TE and MoMH; project administration, TE and MoMH; funding acquisition, TE, KD, MaMH, SH, and MoMH . All authors have read and agreed to the published version of the manuscript.

## Conflict of Interest

The authors declare that the research was conducted in the absence of any commercial or financial relationships that could be construed as a potential conflict of interest.

## Publisher’s Note

All claims expressed in this article are solely those of the authors and do not necessarily represent those of their affiliated organizations, or those of the publisher, the editors and the reviewers. Any product that may be evaluated in this article, or claim that may be made by its manufacturer, is not guaranteed or endorsed by the publisher.

## Abbreviations

*ABCB1*, ATP Binding Cassette Subfamily B Member 1; *ABCC1*, ATP Binding Cassette Subfamily C Member 1; *ABCG2*, ATP Binding Cassette Superfamily G Member 2; ALL, Acute Lymphoblastic Leukemia; AXL, AXL receptor tyrosine kinase; BCRP, Breast Cancer Resistance Protein; *BRAF*, B-Raf proto-oncogene; EMT, Epithelial-Mesenchymal Transition; FAK, Focal Adhesion Kinase; HGF, Hepatocyte Growth Factor; hRFC, Human folate carrier’s; MAPK, Mitogen-Activated Protein Kinase; *MDR1, MDR2*, Multidrug Resistance Gene; MITF, Microphthalmia-associated Transcription Factor; MRP1, Multi-drug Resistance-associated Protein 1; MRP2, Multi-drug Resistance-associated Protein 2; NF-kB, Nuclear factor of kappa light polypeptide gene enhancer in B cell; NSCLC, Non-Small Cell Lung Carcinoma; P-gp, P-glycoprotein; PIK3CA, Phosphatidylinositol-4,5-bisphosphate 3-kinase, catalytic subunit alpha; TKI, Tyrosine Kinase Inhibitor.
